# Clinical and laboratory comparison of severe (Group B and C) Dengue cases with molecular characterization from 2019 epidemics in Dhaka, Bangladesh

**DOI:** 10.1371/journal.pntd.0012686

**Published:** 2024-11-25

**Authors:** Fazle Rabbi Chowdhury, Zazeba Hossain, Nahid Parvez, Forhad Uddin Hasan Chowdhury, Mohammad Anwarul Bari, Sudip Ranjan Deb, Mustak Ibn Ayub, Md Uzzwal Mallik, Sakib Aman, Mohammad Ahsanul Haque, Md Arman Hossain, Murada Alam, Muhammad Kamrul Islam, Md Mujibur Rahman

**Affiliations:** 1 Department of Internal Medicine, Bangabandhu Sheikh Mujib Medical University, Dhaka, Bangladesh; 2 Department of Tropical Medicine, Mahidol-Oxford Tropical Medicine Research Unit (MORU), Bangkok, Thailand; 3 Department of Genetic Engineering and Biotechnology, Dhaka University, Dhaka, Bangladesh; 4 Department of Medicine, Dhaka Medical College and Hospital, Dhaka, Bangladesh; 5 Department of Medicine, Sir Salimullah Medical College and Mitford Hospital, Dhaka, Bangladesh; 6 Department of Medicine, Mugda Medical College and Hospital, Dhaka, Bangladesh; The University of Hong Kong, CHINA

## Abstract

Acute arboviral infections like dengue have a significant negative socioeconomic and health impact on many tropical and subtropical areas of the world. About 3.9 billion Individuals are at risk of contracting the dengue virus and Asia bears the brunt of that load. Bangladesh, like other south-east Asian countries faced a massive outbreak of dengue in 2019. This cross sectional study was done in three tertiary care centers in Dhaka, Bangladesh during this worst outbreak of dengue. The study was conducted from 1st July to 31^st^ December, 2019 with an aim to describe the clinical and laboratory variations among severe dengue cases and to conduct a serotype survey. This might help to understand the future changes in the clinical or serological profile of the circulating dengue virus. The study enrolled 1978 participants who were grouped into group B (Patients with warning signs or risk factors who should be admitted for close observation as they approach critical phase) and C (Patients having severe plasma leakage leading to dengue shock and/or fluid accumulation with respiratory distress, severe organ impairment and severe metabolic abnormalities) according to national guidelines. Furthermore, 81 samples were serotyped using Qiagen One step RT-PCR kit (Cat. No: 210212). In addition sequencing (ABI sequencing platform) of partial C-prM gene of five DENV-3 isolates were done and analyzed (BLAST tool of NCBI) for phylogenetics (MEGA6 software package). Among the 1978 enrolled participants group B and C patients were 1580 (80%) and 398 (20%) respectively. The median (IQR) age of the patients were 26(11 to 41). Maximum proportion of the enrolled were male (72.3%) (p = 0.0002). Most common co- morbidities were hypertension (90; 4.5%) and DM (70; 3.5%). Group C patients more commonly presented with vomiting (p 0.133), diarrhea (p<0.0001) and abdominal pain (p 0.0203). The common mode of bleeding was melaena (12%). Thirteen (0.7%) patients succumbed to death, 12 of them belonged to group B who mostly presented with GI manifestations (99.9%) and melaena (12%). Only 5.6% of the cases were secondarily infected. In group C cases low hemoglobin and hematocrit was observed with high AST (p 0.004, 0.006 and 0.0016 respectively). Fluid requirement was also more in the same group (p<0.0001). Group B patients had a higher platelet requirement (p = 0.0070). Twelve patients (0.7%) required ICU. The management profile of these cases are showcased here which highlights minimal use of antibiotics and no application of steroids, which abides by the current national protocol. Furthermore, 81 samples from enrolled participants were serotyped and majority (79%) yielded DENV-3, followed by DENV 2 & 3 co-infection (13.6%) and DENV-2 alone (7.4%). Following phylogenetic analysis DENV-3 and DENV2 were deemed to be of genotype I and cosmopolitan variety respectively. This study presents the first instance of heterogeneous co-infection with several serotypes since 2000 in Bangladesh. It also gives an overview of serotype prevalence, management evaluation and clinical results that promises to navigate future control planning.

## Author summary

Bangladesh experienced an unprecedented dengue surge in 2019. This cross sectional study was done in 3 hospitals in Dhaka, Bangladesh spanning from 1st July to 31^st^ December, 2019 enrolling a total of 1978 participants. We observed a shift of clinical presentations and varied laboratory alterations in the severe groups which was portrayed according to national guideline case definitions. Most common co- morbidities were hypertension (4.5%). The major mode of bleeding was melaena (12%). Total 13 (0.7%) patients succumbed to death. The management received by these patients were also elaborated. Although Bangladesh is a hyper endemic region, the serotype survey is rarely done because of logistic and financial constraints. However it has become a burning need to study the infecting serotype of the severe cases to help navigate the control measures and predict the atypical manifestations and also for future vaccine deployment. Here, 81 samples were serotyped and most were serotyped as DENV-3 (79%), followed by DENV 2 (7.4%). Interestingly we also found cases co infected with these two serotypes (13.6%). These strains were sequenced and further molecular analysis were performed. This study presents the first instance of heterogeneous co-infection with several serotypes since 2000 in Bangladesh.

## Introduction

Dengue is the most rapidly spreading mosquito-borne viral illness transmitted to humans by infected *Aedes* mosquitoes. Principally by *Aedes aegypti* and less often by *Aedes albopictus* [1]. It belongs to Flaviviridae family containing closely related four serotypes (DENV 1–4) which are further classified into different genotypes. These serotypes can co-circulate and many countries are hyper endemic for all four serotypes. Recovery from one serotype provides lifelong immunity against that serotype only. Although there is transitory and partial cross-immunity due to antibody-dependent enhancement (ADE), secondary infection with an additional serotype raises the risk of developing severe dengue [2]. Before 1970 only nine (9) countries had reported severe dengue epidemics, now the disease has spread its endemicity in more than 100 countries worldwide. The number of dengue cases reported to WHO increased over 8 fold over the last two decades [2]. It is predicted that by 2080, 60% of the world population (over 6.1 billion) will be at risk of dengue infection [3].

Bangladesh encountered first official outbreak in 2000 reporting 5551 cases with 1.6% case fatality. Since then there has been cyclical outbreaks every few years [4–7]. Largest dengue upsurge till date was recorded in 2019 globally. That year the Americas reported 3.1 million cases. In Asia, Malaysia followed by Philippines and Vietnam reported 1,31,000, 4,20,000 and 3,20,000 cases respectively [2]. Similar phenomenon reported in Nepal [8]. Bangladesh also faced its highest dengue cases throughout the country that year. About 1,01,354 (one hundred one thousand, three hundred fifty-four) confirmed cases required admission and 164 deaths reported [9]. The blow of dengue in 2019 was not just restricted to endemic cities but was spread nationwide [10].

The multi-annual fluctuations in DF (Dengue Fever) or DHF (Dengue Hemorrhagic Fever) incidence as well as cyclical oscillations of the individual serotypes, where the dominant type is gradually replaced over time, are indicative of the temporal epidemiological pattern of dengue [11]. These shift in serotypes and genotypes may lead to severe dengue outbreaks due to ADE [12].

Despite being frequently afflicted with dengue outbreaks, Bangladesh does not have adequate empirical study on dengue virus serotype detection, severity correlation and molecular evolution. This study was done in 2019 during the first large outbreak in Dhaka, which is known as the epicenter of dengue outbreak [13], with an aim to explore the clinical presentations and outcome of the severe cases along with evaluating the management they received. This study also stepped up to detect the prevalent serotype with its phylogenetic analysis.

## Materials and methods

### Ethical approval and study enrollment profile

Written informed consent was taken from every patient or lawful guardian before enrollment. Counselling was done about the nature of the study and about collection of serum sample. Ethical clearance was obtained from Ethical review committee of Bangladesh Medical Research Council, Mohakhali, Dhaka-1212, Bangladesh (memo no-RP/2019/365).

This cross-sectional study done from 1^st^ July to 31^st^ December, 2019 at three tertiary care government hospitals in Dhaka, Bangladesh namely, Dhaka Medical College and Hospital, Mugda Medical College Hospital and Sir Salimullah Medical College Hospital. The whole study was coordinated by Bangabandhu Sheikh Mujib Medical University (BSMMU). S1 Fig shows the map and enrollment profile of the study sites. Suspected dengue cases (Fever with any two of nausea/vomiting, rash, aches/pain, leucopenia, any warning sign or positive tourniquet test) [14] were screened. Those who had fever for 2–7 days, aged more than 16 years and showed either NS1 antigen positivity or Immunoglobulin M positivity were included in this study. Those who did not wish to participate and were co-infected with any bacterial or parasitic infection were excluded from this study. Patients who were complicated into “Expanded dengue syndrome” [14] were also excluded from this study. After enrollment they were broadly divided into Group B (Patients with warning signs [14] or risk factors who should be admitted for close observation as they approach critical phase) and Group C (Patients having severe plasma leakage leading to dengue shock and/or fluid accumulation with respiratory distress, severe organ impairment and severe metabolic abnormalities) according to national dengue case management guidelines [14]. Primary infection were reported by laboratory evidence of NS1 or Anti dengue IgM RDT (rapid diagnostic test) and secondary infections were declared when patients had Anti dengue IgG RDT positivity. Probable and confirmed dengue cases were reported by antidengue IgM positivity and NS1 antigen detection respectively. Data input, sample collection and tests were done following a predefined protocol detailed in S1 text (S1.1 and 1.2).

### Dengue virus serotyping

Serotyping and genotyping was done in the laboratory of the Department of Genetic Engineering and Biotechnology, University of Dhaka, Bangladesh. Reverse transcriptase based nested PCR was used for serotyping following the manufacturers protocol (Qiagen One step RT-PCR kit—Cat. No:210212). This technique generates DNA of four different sizes specific to each serotype from viral RNA which are visualized by agarose gel electrophoresis. RNA extraction was done using NEB RNA extraction kit (Cat No: T2010S). The steps are elaborated in S1 Text (Section S1.3 and Tables A to F.

### Partial Genome Sequencing and phylogenetic analysis

During the step of RT-PCR of DENV genome a partial fragment from C-prM gene of 511 bp length was amplified. After purification with GeneJET PCR Purification Kit (Catalog number: K0701), Sanger sequencing was performed using ABI sequencing platform to determine nucleotide sequences of the 511 bp long PCR products of five samples (Id:1,2,3, 17 & 14). The chromatograms were viewed in FinchTV software and low-quality reads were trimmed from both ends of each sequence. BLAST (Basic Local Alignment Search Tool) of NCBI (National Center for Biotechnology Information) was utilized to confirm that the sequences belonged to DENV-2 and DENV-3 serotypes. Later, two phylogenetic dendrograms were created for these serotypes. Partial C-prM gene sequences of the isolates of this study were aligned to same sequence of known genotypes reported previously which has been used widely as an alternative to envelope protein gene [15–18]. These sequences were retrieved from the GenBank database of NCBI.

The dataset of DENV-2 and DENV-3 are shown in S1 Text (Section S1.4 and Tables G and H. A total of 57 and 62 sequences were used for the phylogenetic analysis for DENV2 and DENV-3 respectively. Isolates of genotype IV (DENV-3) could not be included due to lack of C-prM sequences of this genotype.

The phylogenetic dendrograms of the C-prM gene were constructed using the maximum-likelihood method with the MEGA6 software package (https://megasoftware.net). Multiple alignment was performed using MUSCLE algorithm [19]. The best substitution model for both DENV-2 and DENV-3 datasets were identified to the Kimura two-parameter model with invariable positions and Gamma distributed rates among sites [20]. Based on the findings of a previous study, the tree was statistically supported by bootstrapping with 500 replicates [21].

To look for common sites of mutation, partial c-prM gene sequences of this study and their closely related ones in the cladograms were aligned to reference genomes of the respective serotype. Jalview software was used to visualize the alignments and find the common mutation sites. Partial C-prM gene sequences of representative five samples were deposited to GenBank database under accession number **(OR726315-19)** (Table K in S1 Text).

### Statistical analysis

Data were analyzed using GraphPad prism version 9.3.1 (San Diego, CA, USA). The categorical variables are showed as frequencies and percentages after comparison with Chi-square test/ Fisher’s exact test (two sided). Continuous variables were compared using Mann-Whitney U test (two tailed). The quantitative data are presented as median ± Inter Quartile Range (IQR) or mean ± (standard deviation) SD. A probability value of p < 0.05 was considered statistically significant.

## Results

### Participant enrollment profile of the study

**Fig 1:**
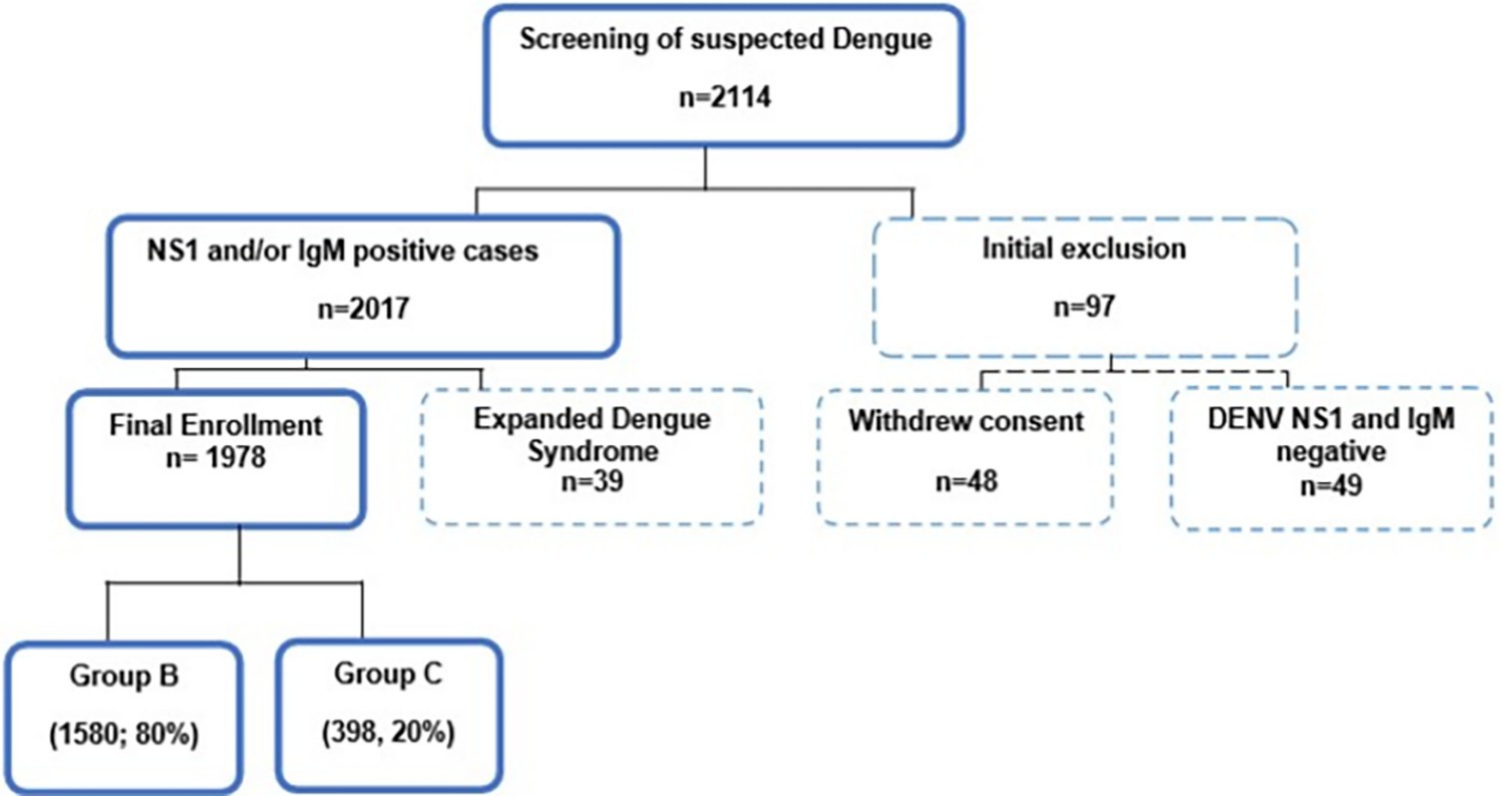
Flowchart showing sequence of patient enrollment. NS1- Non-structural protein 1; IgM- Immunoglobulin M.

A total of 2,114 suspected dengue patients were screened for enrollment and 2,017 met the inclusion criteria (Fig 1). Out of them 39 developed expanded dengue syndrome and thus were excluded. The final enrollment number was 1,978. This cohort were classified into group B (1580, 80%) and group C (398, 20%) according to case definitions following national guideline [14].

### Comparison between baseline characteristics and outcome between group B and group C cases

#### Demographic profile and risk factor

The median (IQR) age of the patients were 26(20 to 35). Maximum proportion of the enrolled were male (72.3%) (p = 0.0002) (Table 1). The cases were mainly residing in Dhaka (1299; 65.7%) although a higher proportion that came from outside Dhaka were observed in group C than group B (37.2% vs 26.8%). Most of the cases were students (542; 27.4%) followed by housewives (535, 26.5%), service holders (311, 15.7%) and others. Hypertension (90; 4.5%) was the most common associated co morbidity along with diabetes mellitus (DM) (70, 3.5%) and chronic kidney disease (CKD) (10; 0.5%).

**Table 1:** Demographic profile and risk factors of dengue cases.

Characteristics:	Total (n = 1978)	Group B (n = 1580)	Group C (n = 398)	p value*
Age (median, IQR)	26, 20–35	27, 20–36	25, 19–35	**0.0053**
Male, n (%)	1430 (72.3)	113 (74.2)	257 (64.6)	**0.0002**
Residence, n (%)				0.1790
Dhaka	1299(65.7)	1049(53)	250(62.8)	
Elsewhere	679(34.3)	531(26.8)	148(37.2)	
Occupation, n (%)				0.7132
Student	542(27.4)	441(27.9)	101(25.4)	
Housewife	525(26.5)	418(26.5)	107(26.9)	
Service holders	311(15.7)	250(15.8)	61(15.3)	
Businessmen	306(15.5)	244(15.4)	62(15.6)	
Co-morbidities, n (%)				0.1950
Hypertension	90(4.5)	78(4.9)	12(3)	
Diabetes mellitus	70(3.5)	56(3.5)	14(3.5)	
Chronic Kidney Disease	10(0.5)	10(0.6)	0(0)	

*Mann-Whitney and Chi-square test (two-sided) was applied to identify the level of significance. P-value <0.05 was considered statistically significant (bold).

### Clinical presentation and outcome

The median duration of fever was 4 days in all groups. The classical presentation of both groups were with headache (67.2%) and bodyache (76.8%). Notably, bodyache was more frequently observed in group B (1276; 80.8%) (p<0.0001). Common mode of bleeding was melaena (235; 12%). Hemorrhagic manifestations were more prevalent in group C cases than group B (167; 42% vs 588; 37.2%). Gum bleeding was observed more in group C than group B (9.5% vs 6; p = 0.0132). Both of the groups showed a high rate of GI manifestations (99.9%) (Table 2). Vomiting, diarrhea and abdominal pain were significantly high in group C (P = 0.0133, <0.0001 and 0.0203 respectively). A total of thirteen patients (0.7%) died which mostly belonged to group B (12, 0.8%) (p = 0.2618). There was no significant difference in the physical signs among both groups, except in vitals which can be explained by the disease course.

**Table 2:** Clinical presentations and outcome of dengue cases.

Characteristics	Total (n = 1978)	Group B (n = 1580)	Group C (n = 398)	p value*
**Duration of fever (Median, IQR)**	4, 2–6	4, 2–6	4,1–6	0.2443
**General symptoms, n(%)**				
Body ache	1519(76.8)	1276(80.8)	243(61.0)	**<0.0001**
Headache	1330(67.2)	1054(66.7)	276(69.3)	0.3392
Rash	326(16.5)	248(15.7)	78(19.6)	0.0694
Retro orbital pain	156(7.9)	212(13.4)	78(19.6)	**0.0025**
**Gastrointestinal manifestations, n(%)**	1977(99.9)	1579 (99.9)	398(100.0)	1.0000
Vomiting	1068(54.0)	831(52.6)	237(59.5)	**0.0133**
Nausea	844(43.0)	690(44.0)	154(38.7)	0.0788
Diarrhea	514(26.0)	373(23.6)	141(35.4)	**<0.0001**
Abdominal pain	284(14.4)	212(13.4)	72(18.0)	**0.0203**
**Hemorrhagic manifestations, n(%)**	755(38.2)	588(37.2)	167(42.0)	0.0836
Melaena	235(12.0)	188(12.0)	47(12.0)	1.0000
Gum bleeding	132(6.7)	94(6.0)	38(9.5)	**0.0132**
Conjunctival bleed	55(2.8)	48(3.0)	7(1.8)	0.2306
Per vaginal bleed	50(2.5)	37(2.3)	13(3.3)	0.2862
Hemoptysis	31(1.6)	22(1.4)	9(2.3)	0.2555
**Signs, n(%)**				
Positive tourniquet test	259(13.0)	196(12.4)	63(15.8)	0.0805
Pleural Effusion	96(4.8)	80(5.0)	16(4.0)	0.4355
Ascites	69(3.5)	59(3.7)	10(2.5)	0.2852
Temperature (Median, IQR)	100, 98–102	100, 98–102	101, 99–103	**0.0001**
Heart rate (Median, IQR)	80, 74–88	80, 72–88	82, 76–92	**0.0002**
Pulse pressure (Median, IQR)	30, 30–40	40, 30–40	20, 20–20	**<0.0001**
**Outcome** ^**^ **, n(%)**				0.2618
Death	13(0.7)	12(0.8)	1(0.3)	

*: Calculated using Mann-whitney (two tailed) and Fisher’s exact test (two-sided)

**: hospital outcome, *p-value <0*.*05 was considered statistically significant (bold)*.

### Hematological and biochemical differences

In group C cases low hemoglobin and hematocrit was observed with high AST (p 0.004, 0.006 and 0.0016 respectively) (Table 3). There was no statistically significant difference between the groups in terms of total WBC count, platelet count, neutrophil lymphocyte ratio, HbA1c, alanine aminotransferase (ALT), serum creatinine, RBS, serum electrolytes, serum calcium and serum lipase.

**Table 3:** Comparison of hematological and biochemical characteristics between the groups (Median, IQR).

Characteristics (units)	Total (n = 1978)	Group B (n = 1580)	Group C (n = 398)	p-value*
Hemoglobin (g/dl)	13.3,12–14.5	13.4,12–14.6	12.9,12–14.3	**0.0040**
Hematocrit (%)	40,36.7–43	40.1,37–43	39,36–43	**0.0060**
Total count WBC (/μL)	5000,3900–7000	5045,3993–7006	5000,3800–7000	0.4849
Platelet Count (/L)	104500,59000–156000	103000,59000–156000	106000,60000–155000	0.6563
N:L ratio	1.8,1–3.2	1.8,1–3.1	2,1.1–3.6	0.1057
AST (U/L)	100,61.3–167.3	93,58–150	130,93–200	**0.0016**
ALT(U/L)	62.5,40–110	61,40–106.8	66,43–130.5	0.9240
Serum creatinine (μmol/L)	1, 0.9–1.2	1, 0.9–1.2	1, 0.8–1.1	0.4300
RBS (mmol/L)	6.9,5.8–8.7	7,5.9–8.8	6.5,5.4–8.1	0.2505
HbA1c (%)	7.2,7.2–8.4	7.2,7.2–8.7	7,6.7–7.6	0.4545
Serum Sodium (mmol/L)	137,134–140	137,134–140	136,133.8–139.3	0.5813
Serum Potassium (mmol/L)	4,3.7–4.5	4,3.7–4.5	4,3.6–4.8	0.4765
Serum Chloride (mmol/L)	101,98–103	101,98–103	101,98–105	0.3898
Serum Bicarbonate (mmol/L)	24,20–28	24,20.5–27.5	24,20–28	0.2634
Serum Calcium (mmol/L)	8,7.4–8.9	8,7.5–8.6	8.5,7.3–9.3	0.3966
Serum Lipase (mmol/L)	222.5,145–484.3	273,157.2–615.5	199,110.5–210	0.2253
Secondary infection**, n(%)	110 (5.6)	89 (5.6)	21 (5.3)	0.9026

*Mann-Whitney test was applied to identify the level of significance

**Secondary infection concluded by Antidengue IgG positivity

p-value <0.05 was considered statistically significant (bold).

### Comparison of management received by two groups

Enrolled patients were admitted for a median (IQR) of 5 (4 to 6) days (Table 4). The only statistically significant difference in management in both groups were in total fluid requirement and platelet transfusion. Patients in group C needed more IV fluids (5 vs 4 L) than group B (P<0.0001). Platelet transfusion was done more in Group B patients compared to Group C (2.8% vs 1.3%) (p = 0.0070). Most patients were managed by crystalloids (1694; 85.6%) and only 14 patients (0.7%) needed colloids. However, around 5% patients required both (P = 0.7872). Transfusion of fresh whole blood and FFP was done in 5.3% (104) and 0.05% (1) cases respectively. The median (IQR) requirement of blood was of 2 (1 to 2) units (P = 0.6510) which was indifferent in both groups. Only one patient (0.05%) received antibiotic in group B. No one got steroids in any of the three study sites. Only 13 (0.7%) of the patients needed ICU support mostly belonging to group B (11, 0.7%) (p = 0.6691).

**Table 4:** Management delivered to both groups during the 2019 outbreak.

Characteristics (Median, IQR)	Total (n = 1978)	Group B (n = 1580)	Group C (n = 398)	p-value*
Days of hospital stay(days)	5,4–6	5,4–6	5,4–7	0.8499
Total IV Fluid requirement (L)	4.5,2–6	4,2–6	5,3–7	**<0.0001**
Units of whole blood transfusion (L)	2,1–2	2,1–2	2,1–2	0.0651
Need of IV fluid, n (%)	1908(96.5)	1501(95)	395(99.2)	0.0518
Type of IV fluid given				0.7872
Crystalloid, n (%)	1694(85.6)	1342(85)	352(88.4)	
Colloid, n (%)	14(0.7)	11(0.7)	3(0.8)	
Both, n (%)	97(4.9)	74(4.7)	23(5.8)	
Blood transfusion, n (%)	104(5.3)	82(5.2)	22(5.5)	0.6510
Apheresis platelet transfusion, n (%)	49(2.5)	44(2.8)	5(1.3)	**0.0070**
Fresh frozen plasma, n (%)	1(0.1)	1(0.1)	0(0.0)	0.6157
Use of antibiotics, n (%)	1(0.1)	1(0.1)	0(0.0)	0.6157
Use of steroid, n (%)	0(0.0)	0(0.0)	0(0.0)	N/A
ICU support, n (%)	13(0.7)	11.0(0.7)	2(0.5)	0.6691

*Mann-Whitney U test (two tailed) and Chi-square test (two-sided) was applied to identify the level of significance. p-value <0.05 was considered statistically significant (bold).

### Dengue serotyping and comparison of manifestations

**Fig 2:**
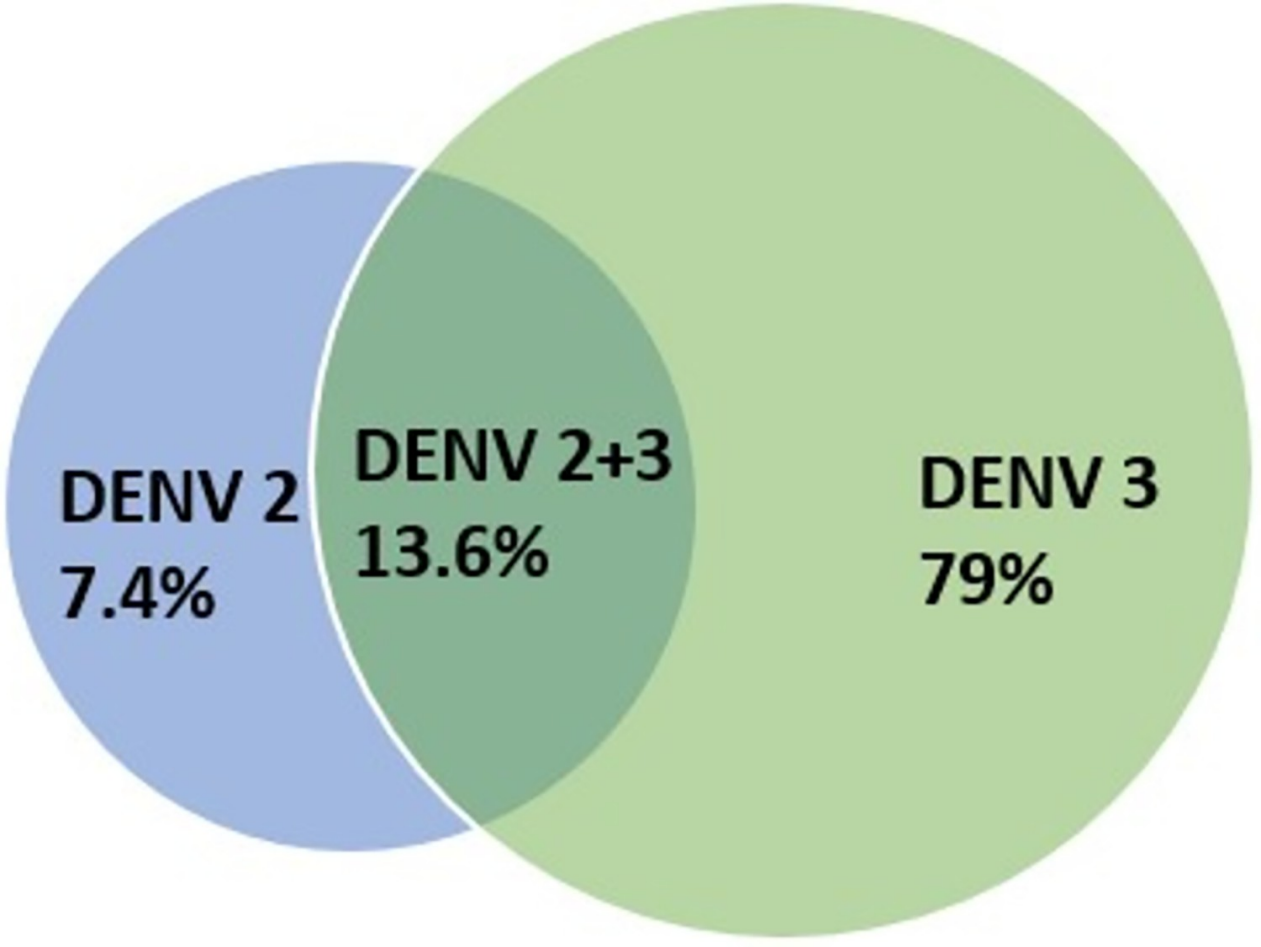
Venn diagram showing percentage of prevalent serotypes during 2019 outbreak in Dhaka, Bangladesh.

Among 86 extracted RNA samples 81 was successfully serotyped out of which DENV-3, DENV-2 and co infection with both DENV-2 and 3 were detected in 64 (79%), 6 (7.4%) and 11 (13.58%) samples respectively (Fig 2). The gel electrophoresis images of samples, from which serotypes were identified are provided in S2 Fig. Patients who were found to have DENV-3 infection or co-infection (DENV-2 &3) mostly fell into group B (>60%). On the other hand, DENV-2 was identified in equal cases of group B and Table I in S1 Text (p = ns).

Features like headache (66.7%), myalgia (50%), joint pain (50%), retro orbital pain (33.3%), and rash (33.3%) were the common complaints in these cases more frequently seen in co infected patients (DENV-2 &3) (Table J in S1 Text). Lethargy was a significant presentation in co infection group (p = 0.0314). The classical dengue symptoms like rash and retro orbital pain were only observed in about one third cases in DENV-2 and DENV-3 infections. Bleeding manifestations were more common in DENV-2 than DENV-3 (66.7% vs 51.6%). Whereas, GI manifestations were more common in DENV-3 than DENV-2 (92.2% vs 83.3%). Major mode of haemorrhage in these cohort was GI bleeding in the form of melaena (66.7%), especially in DENV-2 cases. Although negligible, genitourinary bleeds (per vaginal and hematuria) were reported in co infected cases. DENV-3 infected cases presented with bleeding involving almost all systems (including hemoptysis, gum bleeds, epistaxis, IV puncture bleeds etc). Although these differences were not statistically significant.

### Sequence analysis and phylogenetic relationship

In addition to serotyping, the nucleotide sequences of 511 bp fragment of partial C-prM gene of sample 1, 2,3,17 and 14 were identified (GenBank accession number OR726315-19 respectively). The sequences are listed in Table K in S1 Text. NCBI BLAST was utilized on samples 1, 2, 17 and 14 (sample 3 was excluded due to small sequence size) to confirm whether these sequences belonged to serotypes determined by PCR and Gel Electrophoresis.

Regarding DENV-3 serotype, the sequence from Sample no 1(OR726315) showed 99.43% identity with DENV-3 isolates that were reported previously to circulate in Dhaka city during the year 2017 [15]. Sample no 2(OR726316) was found to be identical (100% similarity) with isolates (MW599418.1, MW599415.1) prevalent during 2019 outbreak in Dhaka and Rangpur city of Bangladesh [18]. The sequence from sample no. 17(OR726318) exhibited 100% identity with two GenBank entries (MN922033.1, MN922034.1) of DENV-3 that were isolated in China from Bangladeshi travelers in the year 2019. As for DENV-2 serotype, sample no. 14(OR726319), 99.74% percent identity with three DENV-2 isolates (LC436675.1, LC436674.1, LC436673.1) was observed and all of these were reported to be present in 2017 outbreak in Bangladesh [15].

**Fig 3:**
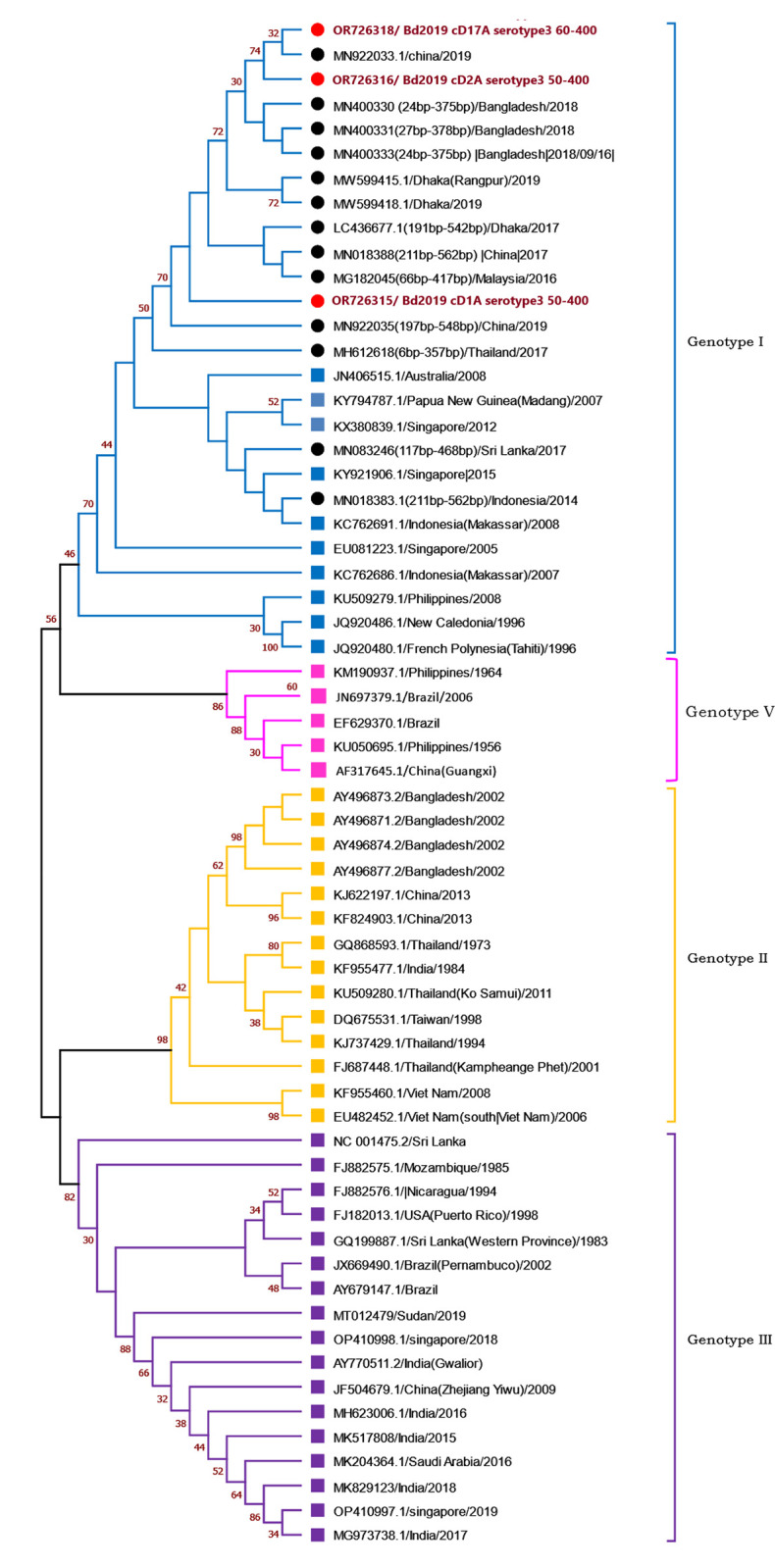
Dendrogram of partial C-prM gene sequences of different DENV-3 isolates. Red circle—sequences determined in this study, Black circle—sequences from BLAST search with no previous report about genotype. Squares: Sequences retrieved from GenBank with reports about genotypes in previous studies,[15–17]. Bootstrapping values larger than 30 are shown at the respective nodes. Respective genotypes are shown on the right side.

**Fig 4:**
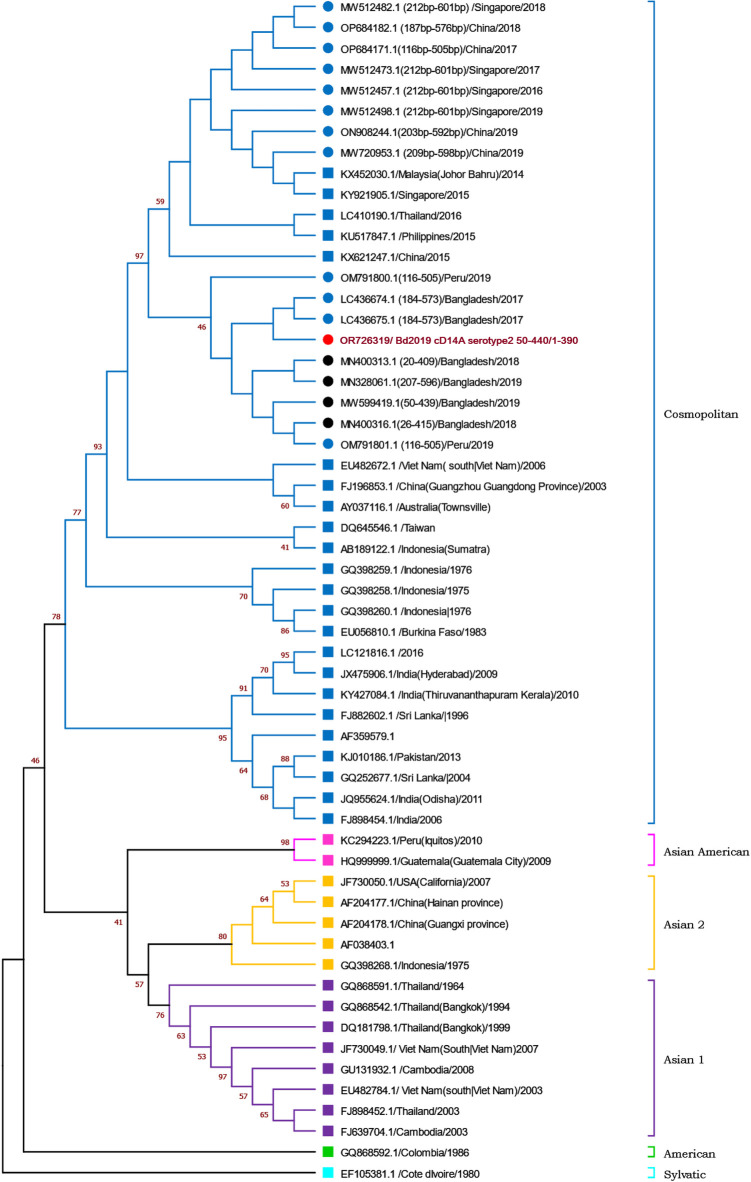
Dendrogram of partial C-prM gene sequences of different DENV-2 isolates. Red circle—sequences determined in this study, Black circle—sequences from BLAST search with no previous report about genotype. Blue circle—sequences from BLAST search that were reported as Cosmopolitan genotype. Squares: Sequences retrieved from GenBank with reports about genotypes in previous studies,[15–17]. Bootstrapping values larger than 30 are shown at the respective nodes. Respective genotypes are shown on the right side.

Partial C-prM gene sequences were utilized to create two separate dendrograms for each serotype. The findings were consistent with previously reported genotypes where envelope gene (E-gene) sequence was used. The DENV-3 isolates obtained in this study belong to genotype 1 as in the dendrogram, these isolates can be found clustered around the clades previously reported to be of genotype I, which is in consensus with the sequences obtained from BLAST search. **(Fig 3)**. In the case of DENV-2 dendrogram, the isolate of this study was found to be in the clade of Cosmopolitan genotype **(Fig 4)**. Moreover, it was found to very closely related to DENV-2 isolates that were collected from Bangladesh during the year of 2017, 2018, 2019. Among them, the sequences of 2017 have already been reported to of Cosmopolitan genotype.

## Discussion

Predominance in male (72.3%) cases was observed in this study which was a constant observation in previous outbreaks [22–24] (Table 1). This male dominance can be explained by restrictions of health seeking behavior in woman (probability is 1.73 times higher in male) [25] due to cultural and socioeconomic factors [26,27]. Only two studies, from 2011 (54%) and 2019 (57.1%), have reported female dominance [24,28]. The median (IQR) age of this cohort was 26(20 to 35). Since the outbreak of 2000, majority reports showed similar younger age distribution which may be related to their more outdoor activities [22,23,29–31]. However, in 2011 Islam et al. [28] reported a mean age of 46 years (SD 28 to 64). A few studies of 2019 have also reported prevalence in older age group (mean: 37–38 years) [24,32]. Which is in line with the global burden of disease study 2019 showing an increasing trend of incidence in elderly [33]. The occupation background and associated co-morbidities of the cases were similar to last outbreak [31,34]. Most of the cases who came from outside Dhaka fell into group C (Table 1) or dengue shock syndrome (DSS). This might indicate a delay in adequate management in this group since the cases were referred from other cities.

This study shows a decline in cutaneous manifestations from 55% to 16.5% and bleeding manifestations from 46% to 38.2% from previous studies [22,35]. Sub-conjunctival bleeds were more common (20%) in 2016 [23] but this study reported more instances of melaena (12%)(Table 2). Similar pattern was seen in 2002 (50% GI bleeds) when DENV-3 was predominant [29]. Arifuzzaman et al. reported that patients having deranged transaminases had more chance of bleeding morbidities [35]. In this study a higher AST in patients with group C (p 0.0016) or DSS been observed. Although more haemorrhagic manifestations were reported in group B. Only 5.6% of the cases were secondary infection in this cohort (Table 3). The major infecting serotypes, DENV-3 and DENV-2 was already reported to be more pathogenic and associated with severe manifestations [36,37]. This might explain the increased number of severe cases in 2019. This also bell an alarm that in near future, exposure of Dhaka residents to a different strain could bring more severe outbreaks with increased number of severe cases and death.

Concurrent infection with multiple serotypes can be transmitted by a single *Aedes aegypti* mosquito harboring multiple serotypes or multiple bites from different mosquitoes. Antibody dependent enhancement (ADE) can enhance the susceptibility to heterotypic infection as it can promote coexistence of strains that alters the dynamics of presentations [12,38]. This may be the reason behind increase prevalence of severe manifestations like fluid leakage and other warning signs in these cases [39]. However, there are other studies stating that mono infection and co infections have indifferent presentations [40]. According to previous reports primary infection with DENV-3 and secondary infection with DENV-2, DENV-3 and DENV-4 showed increased the risk of severe dengue [41]. Nisalak et al conducted a study in Thailand for near about three decades and found that DENV-3 predominance was responsible for severe outbreak years and DENV-2 was responsible for most of the secondary dengue infections and DHF [11]. In Bangladesh DENV-3 resurfaced after nine years in 2017 from the first dengue case detection in 1964 [7,42,43]. During the four decade period of its predominance, only one massive outbreak took place in 2000. Back then concurrent infection (12.5%) with DENV-2 and DENV-4 with DENV-3 was recorded [4]. After 2000 outbreak, this study of 2019 reports one of the first evidence of concurrent infection with multiple serotypes (DENV-2 and 3) (Fig 2) and their clinical profile from three tertiary care centers of Dhaka city. Although from previous report it is known that DENV-3 Genotype II has been circulating in the continent [29], this study marks the presence of DENV-3 (genotype-I) which is in line with Titir et al.[18]. Although whether there has been a complete shift of the strain that has resulted this mass outbreak needs much more exhaustive sero-surveillance.

This study shows no significant difference of infecting serotype amongst Group B and C cases (Table I in S1 Text). In this cohort, DENV-3 mostly presented with GI complaints which is in line with Halsey et al. [44]. Previous studies recorded that joint pain are common presentations of DENV-3 and DENV-2 [44,45]. This interesting shift of symptoms (musculoskeletal to gastro-intestinal) in recent outbreaks is a future area of research. Significant relationship of low platelet count with DENV-2 was also reported previously [45] but we only found higher frequency of bleeding manifestations (Table J in S1 Text). The shifts in clinical presentations can also be observed in other countries. In India, DENV-3 was a predominant infecting serotype in 2004 and from 2016 to 2018[46–48]. Interestingly, increase in GI symptoms were prevalent around those times with spike in severe dengue cases [49,50]. Similar pattern can be seen in Sri Lanka during 2017 to 2018[51] when in late 2016 DENV-3 was found co circulating with dominant DENV-2(cosmopolitan)[52]. Although ADE is a factor that enhances heterotypic infection by promoting coexistence of multiple serotypes [12,38], most of the cases in this study was of primary infection as per RDT reports. Unfortunately, further correlation with antibody titres were not done due to poor resource setting.

The detected DENV-3 (Genotype I) isolate (OR726315) was found in closer relationship with isolates from 2017 (Dhaka) and 2019 (Dhaka and Rangpur) from Bangladesh. It was also found to be related to one isolate from China from 2019 which was collected from Bangladeshi travelers (Fig 3). DENV-2 (Cosmopolitan) isolate identified in this study showed connection with reported isolates of 2017–2019 from the same region. It may also be related to isolates of Peru from 2019 (Fig 4). This suggests there were no major molecular shift in these isolates from 2017 to 2019.

The recent studies and national case management guideline recommend against using steroids and unnecessary antibiotics in dengue as there is no evidence of usefulness [53]. In this cohort of 1978 people only one Group B case received antibiotics and none of them received steroids (Table 4). This picture ascertains prompt adherence to the national guideline in 2019 outbreak in these study sites [14]. ICU transfers and mortality rate was also minimal than other hospital reports of the same outbreak [32].

The mortality rate, as reported by DGHS (Director General Health Services), Bangladesh press release, is on uptrend from 0.16% in 2019 to 0.36% in 2021. In the year 2022, it has levelled up to 0.45% [9]. This number is just the tip of the ice berg due to the passive surveillance system and inadequate lab facilities. After, 2019 outbreak there has been a revision in national guideline and government has ensured proper training to health care providers to ensure timely treatment.

In this study, serotyping of all the samples could not be done due to low resources. More engaging studies involving other regional areas need to be done in light of this work for better understanding of viral evolution and its correlation with clinical and laboratory dynamics. This way we can probably anticipate the nature of future outbreaks and detect loopholes in the tackling system.

## Conclusion

In order to forecast the severity of an outbreak in a hyperendemic country like Bangladesh, type-specific DENV records must be documented. Because of global trade, increased cross country mobility, and rapid and unplanned urbanization emergence of novel strains and co-circulation of several strains are a significant problem in Bangladesh. Focused measures need to be taken to enhance laboratory capacity, strengthening sero-surveillance and reducing the out-of-pocket expenditure by implementing guideline based management and evaluation.

## Supporting information

S1 Fig Enrollment profile of three study sites in Dhaka, Bangladesh. The red triangle represents the study co-ordinator site. The orange, blue and green circle depicts three data collection points. Site wise number of enrollment (black brackets) and percentage of group B and group C (purple and red respectively) are also shown. Map was plotted using Datawrapper website (available at https://www.datawrapper.de/_/2ew1H/). Basemap shapefile was extracted from OpenStreetMap with due permission from the concerned. (Open Database License, ODbL 1.0, see https://www.openstreetmap.org/copyright").
(TIF)

S2 Fig **(S2A and S2B):** Figure showing specimens from gel electrophoresis of DNA fragments generated at step 3. Fragment size of 290bp and 119bp suggests presence of DENV-3 and DENV-2 respectively. Concurrent infection by DENV-2 and DENV-3 can also be seen in S2B Fig. Presence of no other serotype was identified.
(TIF)

S1 Text Additional supplement of methodology and results including supporting information Tables A to K. Table A: List of primers used in PCR reactions. Table B: Reaction condition for reverse transcriptase PCR. Table C: Reagent composition for reverse transcriptase PCR. Table D: Reaction condition of nested PCR. Table E: Reagent composition of Nested PCR. Table F: Serotypes and respective DNA fragment sizes. Table G: List of DENV-2 sequences used in the dendrogram. Table H: List of DENV-3 sequences used in the dendrograms. Table I: Serotype distribution between group B and C cases out of 81. Table J: Description of clinical presentations and laboratory parameters of 81 cases according to serotype distribution during the 2019 outbreak. Table K: Partial sequence of sample 1, 2, 3, 17 & 14.
(DOCX)

## References

[1] WHO/TDR. Dengue: guidelines for diagnosis, treatment, prevention and control—New edition. WHO Libr Cat Data Dengue. 2009;41: 29–29. doi: 10.1176/pn.41.1.0029b 23762963

[2] Dengue and severe dengue. [cited 31 Jul 2021]. Available: https://www.who.int/news-room/fact-sheets/detail/dengue-and-severe-dengue

[3] Messina JP, Brady OJ, Golding N, Kraemer MUG, Wint GRW, Ray SE, et al. The current and future global distribution and population at risk of dengue. Nat Microbiol. 2019;4: 1508–1515. doi: 10.1038/s41564-019-0476-8 31182801 PMC6784886

[4] Aziz MM, Hasan KN, Hasanat MA, Siddiqui MA, Salimullah M, Chowdhury AK, et al. Predominance of the DEN-3 genotype during the recent dengue outbreak in Bangladesh. Southeast Asian J Trop Med Public Health. 2002;33: 42–48. 12118459

[5] Ahmed AKMM, Alam N, Sultana S. Circulating dengue virus serotypes in Bangladesh from 2013 to 2016. VirusDisease. 2018; 3–7. doi: 10.1007/s13337-018-0469-x 30159364 PMC6111961

[6] Akther T, Muraduzzaman AKM, Parvin S, Tabssum S, Munshi S. Molecular & serological study of dengue virus-infected patients attending a tertiary hospital of Dhaka city, Bangladesh (2013 to 2016). Indian J Med Res. 2019;150: 96. doi: 10.4103/IJMR.IJMR_738_18 31571636 PMC6798611

[7] Rahim R, Hasan A, Hasan N, Nakayama EE, Shioda T, Rahman M. Diversity of Dengue Virus Serotypes in Dhaka City: From 2017 to 2021. Bangladesh J Med Microbiol. 2021;15: 23–29. doi: 10.3329/bjmm.v15i2.57817

[8] Rijal KR, Adhikari B, Ghimire B, Dhungel B, Pyakurel UR, Shah P, et al. Epidemiology of dengue virus infections in Nepal, 2006–2019. Infect Dis Poverty. 2021;10: 1–10. doi: 10.1186/s40249-021-00837-0 33858508 PMC8047528

[9] Directorate General of Health Services (DGHS) | Government of Bangladesh. Daily Dengue Status Report: 2019. 2019. Available: https://old.dghs.gov.bd/index.php/bd/home/81-english-root/5200-daily-dengue-status-report

[10] Hossain MS, Siddiqee MH, Siddiqi UR, Raheem E, Akter R, Hu W. Dengue in a crowded megacity: Lessons learnt from 2019 outbreak in Dhaka, Bangladesh. PLoS Negl Trop Dis. 2020;14: e0008349. doi: 10.1371/JOURNAL.PNTD.0008349 32817678 PMC7444497

[11] Nisalak A, Endy TP, Nimmannitya S, Kalayanarooj S, Thisayakorn U, Scott RM, et al. Serotype-specific dengue virus circulation and dengue disease in Bangkok, Thailand from 1973 to 1999. Am J Trop Med Hyg. 2003;68: 191–202. 12641411

[12] Recker M, Blyuss KB, Simmons CP, Hien TT, Wills B, Farrar J, et al. Immunological serotype interactions and their effect on the epidemiological pattern of dengue. Proc R Soc B Biol Sci. 2009;276: 2541–2548. doi: 10.1098/rspb.2009.0331 19369266 PMC2684681

[13] Organization WH. Dengue–Bangladesh. In: Disease Outbreak News; Dengue–Bangladesh [Internet]. 2022. [cited 20 Dec 2022]. Available: https://www.who.int/emergencies/disease-outbreak-news/item/2022-DON424

[14] Revised guideline for clinical management of dengue. [cited 1 Aug 2021]. Available: https://dghs.gov.bd/index.php/en/home/5431-revised-guideline-for-clinical-management-of-dengue

[15] Suzuki K, Phadungsombat J, Nakayama EE, Saito A, Egawa A, Sato T, et al. Genotype replacement of dengue virus type 3 and clade replacement of dengue virus type 2 genotype Cosmopolitan in Dhaka, Bangladesh in 2017. Infect Genet Evol. 2019;75: 103977. doi: 10.1016/j.meegid.2019.103977 31351235

[16] Wittke V, Robb TE, Thu HM, Nisalak A, Nimmannitya S, Kalayanrooj S, et al. Extinction and Rapid Emergence of Strains of Dengue 3 Virus during an Interepidemic Period. Virology. 2002;301: 148–156. doi: 10.1006/viro.2002.1549 12359455

[17] Eldigail MH, Abubaker HA, Khalid FA, Abdallah TM, Musa HH, Ahmed ME, et al. Association of genotype III of dengue virus serotype 3 with disease outbreak in Eastern Sudan, 2019. Virol J. 2020;17: 1–8. doi: 10.1186/S12985-020-01389-9/FIGURES/2 32731875 PMC7392696

[18] Titir SR, Paul SK, Ahmed S, Haque N, Nasreen SA, Hossain KS, et al. Nationwide distribution of dengue virus type 3 (Denv-3) genotype i and emergence of denv-3 genotype iii during the 2019 outbreak in bangladesh. Trop Med Infect Dis. 2021;6. doi: 10.3390/tropicalmed6020058 33919249 PMC8167647

[19] Edgar RC. MUSCLE: multiple sequence alignment with high accuracy and high throughput. Nucleic Acids Res. 2004;32: 1792–1797. doi: 10.1093/nar/gkh340 15034147 PMC390337

[20] Kimura M. A simple method for estimating evolutionary rates of base substitutions through comparative studies of nucleotide sequences. J Mol Evol. 1980;16: 111–120. doi: 10.1007/BF01731581 7463489

[21] Pattengale ND, Alipour M, Bininda-Emonds ORP, Moret BME, Stamatakis A. How Many Bootstrap Replicates Are Necessary? https://home.liebertpub.com/cmb. 2010;17: 337–354. doi: 10.1089/cmb.2009.0179 20377449

[22] Rahman M, Rahman K, Siddque AK, Shoma S, Kamal AHM, Ali KS, et al. First Outbreak of Dengue Hemorrhagic Fever, Bangladesh. Emerg Infect Dis. 2002;8: 738. doi: 10.3201/eid0807.010398 12095447 PMC2730336

[23] Pervin M, Sweety AA, Hossain MZ, Sharmin R, Fatema N, Rahman MA, et al. Sero-epidemiology of Dengue Virus Infection in Clinically Suspected Patients Attended in Dhaka Medical College Hospital During January to December 2016. J Dhaka Med Coll. 2018;26: 111–116. doi: 10.3329/jdmc.v26i2.38825

[24] Ali M, Acherjya GK, Islam AKMM, Alam ABMS, Rahman SMS, Chowdhury R Sen, et al. Clinical profile, haematological changes and outcomes of dengue patients in dengue outbreak-2019 in Jashore, Bangladesh—An observational study. J Med. 2021;22: 33–40. doi: 10.3329/jom.v22i1.51389

[25] Ahmed SM, Adams AM, Chowdhury M, Bhuiya A. Gender, socioeconomic development and health-seeking behaviour in Bangladesh. Soc Sci Med. 2000;51: 361–371. doi: 10.1016/s0277-9536(99)00461-x 10855923

[26] Wang J, Fei Y, Shen H, Xu B. Gender difference in knowledge of tuberculosis and associated health-care seeking behaviors: A cross-sectional study in a rural area of China. BMC Public Health. 2008;8. doi: 10.1186/1471-2458-8-354 18842127 PMC2577657

[27] Ahmed SM, Adams AM, Chowdhury M, Bhuiya A. Changing health-seeking behaviour in Matlab, Bangladesh: do development interventions matter? Health Policy Plan. 2003;18: 306–315. doi: 10.1093/heapol/czg037 12917272

[28] Islam A, Bashar J, Rahim MA, Uddin KN. Severity of hepatitis & it’s correlation with dengue hemorrhagic fever: Experience from a tertiary care hospital. Bangladesh Crit Care J. 2020;8: 76–80. doi: 10.3329/bccj.v8i2.50023

[29] Islam MA, Ahmed MU, Begum N, Chowdhury NA, Khan AH, Parquet M del C, et al. Molecular characterization and clinical evaluation of dengue outbreak in 2002 in Bangladesh. Jpn J Infect Dis. 2006;59: 85–91. 16632907

[30] Islam QT, Basher A, Amin R. Dengue: A practical experience of medical professionals in hospital. J Med. 2012;13: 160–164. doi: 10.3329/jom.v13i2.12751

[31] Hasan MJ, Tabassum T, Sharif M, Khan MAS, Bipasha AR, Basher A, et al. Clinico-epidemiologic characteristics of the 2019 dengue outbreak in Bangladesh. Trans R Soc Trop Med Hyg. 2021;115: 733–740. doi: 10.1093/trstmh/traa126 33190156

[32] Rahman MA, Habiba Begum MU, Md Nizam Uddin H, Monira S, Millat MB. Clinicopathological Profile and Outcome of Dengue Fever: A Tertiary Care Hospital Experience. J Bangladesh Coll Physicians Surg. 2021;39: 213–219. doi: 10.3329/jbcps.v39i4.55941

[33] Du M, Jing W, Liu M, Liu J. The Global Trends and Regional Differences in Incidence of Dengue Infection from 1990 to 2019: An Analysis from the Global Burden of Disease Study 2019. Infect Dis Ther. 2021;10: 1625–1643. doi: 10.1007/s40121-021-00470-2 34173959 PMC8234762

[34] Hossain MZ, Sultana N, Sweety AA, Mahmud R, Khan MMH, Rahman MF, et al. The Predictors of the Severity of Dengue Fever: A Cross-Sectional Study in a Tertiary Care Center of Bangladesh. J Dhaka Med Coll. 2021;29: 77–82. doi: 10.3329/jdmc.v29i1.51175

[35] Arifuzzaman M, Amiruzzaman, Hossain MS, Islam N, Noman A Al, Baroi B, et al. Liver Function Derangement and Bleeding Morbidity in Dengue Fever and Dengue Haemorrhagic Fever in a tertiary Hospital in Bangladesh. Bangladesh J Med. 2021;33: 44–51. doi: 10.3329/bjm.v33i1.56789

[36] Balmaseda A, Hammond SN, Pérez L, Tellez Y, Saborío SI, Mercado JC, et al. Serotype-specific differences in clinical manifestations of dengue. Am J Trop Med Hyg. 2006;74: 449–456. 16525106

[37] Tsang TK, Ghebremariam SL, Gresh L, Gordon A, Halloran ME, Katzelnick LC, et al. Effects of infection history on dengue virus infection and pathogenicity. Nat Commun 2019 101. 2019;10: 1–9. doi: 10.1038/s41467-019-09193-y 30886145 PMC6423047

[38] Ferguson N, Anderson R, Gupta S. The effect of antibody-dependent enhancement on the transmission dynamics and persistence of multiple-strain pathogens. Proc Natl Acad Sci U S A. 1999;96: 790. doi: 10.1073/pnas.96.2.790 9892712 PMC15215

[39] Dhanoa A, Hassan SS, Ngim CF, Lau CF, Chan TS, Adnan NAA, et al. Impact of dengue virus (DENV) co-infection on clinical manifestations, disease severity and laboratory parameters. BMC Infect Dis. 2016;16. doi: 10.1186/s12879-016-1731-8 27514512 PMC4982428

[40] Senaratne UTN, Senaratne UTN, Murugananthan K, Murugananthan K, Sirisena PDNN, Carr JM, et al. Dengue virus co-infections with multiple serotypes do not result in a different clinical outcome compared to mono-infections. Epidemiol Infect. 2020;148. doi: 10.1017/S0950268820000229 32594967 PMC7325333

[41] Soo KM, Khalid B, Ching SM, Chee HY. Meta-analysis of dengue severity during infection by different dengue virus serotypes in primary and secondary infections. PLoS One. 2016;11: 4–14. doi: 10.1371/journal.pone.0154760 27213782 PMC4877104

[42] Aziz MA, Gorham JR, Gregg MB. “Dacca fever”-an outbreak of dengue. Pak J Med Res. 1967;6: 83–92.

[43] Rahman M, Rahim R, Hasan A, Murad AS, Biswas M. Co-circulation of Three Dengue Virus Serotypes in 2017 in Dhaka city: First report from Bangladesh. Bioresearch Commun -. 2022;5: 637–641. Available: https://www.bioresearchcommunications.com/index.php/brc/article/view/72

[44] Halsey ES, Marks MA, Gotuzzo E, Fiestas V, Suarez L, Vargas J, et al. Correlation of Serotype-Specific Dengue Virus Infection with Clinical Manifestations. PLoS Negl Trop Dis. 2012;6. doi: 10.1371/journal.pntd.0001638 22563516 PMC3341333

[45] Yung CF, Lee KS, Thein TL, Tan LK, Gan VC, Wong JGX, et al. Dengue serotype-specific differences in clinical manifestation, laboratory parameters and risk of severe disease in adults, singapore. Am J Trop Med Hyg. 2015;92: 999–1005. doi: 10.4269/ajtmh.14-0628 25825386 PMC4426593

[46] Parveen N, Islam A, Tazeen A, Hisamuddin M, Abdullah M, Naqvi IH, et al. Circulation of single serotype of Dengue Virus (DENV-3) in New Delhi, India during 2016: A change in the epidemiological trend. J Infect Public Health. 2019;12: 49–56. doi: 10.1016/j.jiph.2018.08.008 30274896

[47] Padhi A, Gupta E, Singh G, Parveen S, Islam A, Tarai B. Circulation of DENV-3 Genotype 3 during 2017 to 2018 in Delhi: A Single-Center Hospital-Based Study. J Lab Physicians. 2021;14: 021–026. doi: 10.1055/S-0041-1734017 36186256 PMC9519262

[48] Dash PK, Parida MM, Saxena P, Abhyankar A, Singh CP, Tewari KN, et al. Reemergence of dengue virus type-3 (subtype-III) in India: Implications for increased incidence of DHF & DSS. Virol J. 2006;3. doi: 10.1186/1743-422X-3-55/FIGURES/4 PMC1559593 16824209

[49] Kumar A, Rao CR, Pandit V, Shetty S, Bammigatti C, Samarasinghe CM. Clinical manifestations and trend of dengue cases admitted in a tertiary care hospital, udupi district, karnataka. Indian J Community Med. 2010;35: 386–390. doi: 10.4103/0970-0218.69253 21031102 PMC2963875

[50] Kumar Agrawal V, Saroj Kumar Prusty B, Santosh Reddy C, Krishna Mohan Reddy G, Kumar Agrawal R, Chandra Sekher Srinivasarao Bandaru V, et al. Clinical profile and predictors of Severe Dengue disease: A study from South India. Casp J Intern Med. 2018;9: 334–340. doi: 10.22088/cjim.9.4.334 30510647 PMC6230463

[51] Jayadas TTP, Kumanan T, Arasaratnam V, Gajapathy K, Surendran SN. The clinical profile, hematological parameters and liver transaminases of dengue NS1 Ag positive patients admitted to Jaffna Teaching Hospital, Sri Lanka. BMC Res Notes. 2019;12: 1–5. doi: 10.1186/S13104-019-4655-8/TABLES/3 31547852 PMC6755686

[52] Jayadas TTP, Kumanan T, Gomes L, Jeewandara C, Malavige GN, Ranasinghe D, et al. Regional variation in dengue virus serotypes in sri lanka and its clinical and epidemiological relevance. Diagnostics. 2021;11: 2084. doi: 10.3390/diagnostics11112084 34829432 PMC8618005

[53] Panpanich R, Sornchai P, Kanjanaratanakorn K. Corticosteroids for treating dengue shock syndrome. Cochrane database Syst Rev. 2006 [cited 13 Jun 2023]. doi: 10.1002/14651858.CD003488.pub2 16856011

