# Supplementary figures and images for: Clinical and laboratory comparison of severe (Group B and C) Dengue cases with molecular characterization from 2019 epidemics in Dhaka, Bangladesh
Source: PLoS Negl Trop Dis. 2024 Nov 25;18(11):e0012686. doi: 10.1371/journal.pntd.0012686 (PMC11627391; doi:10.1371/journal.pntd.0012686)

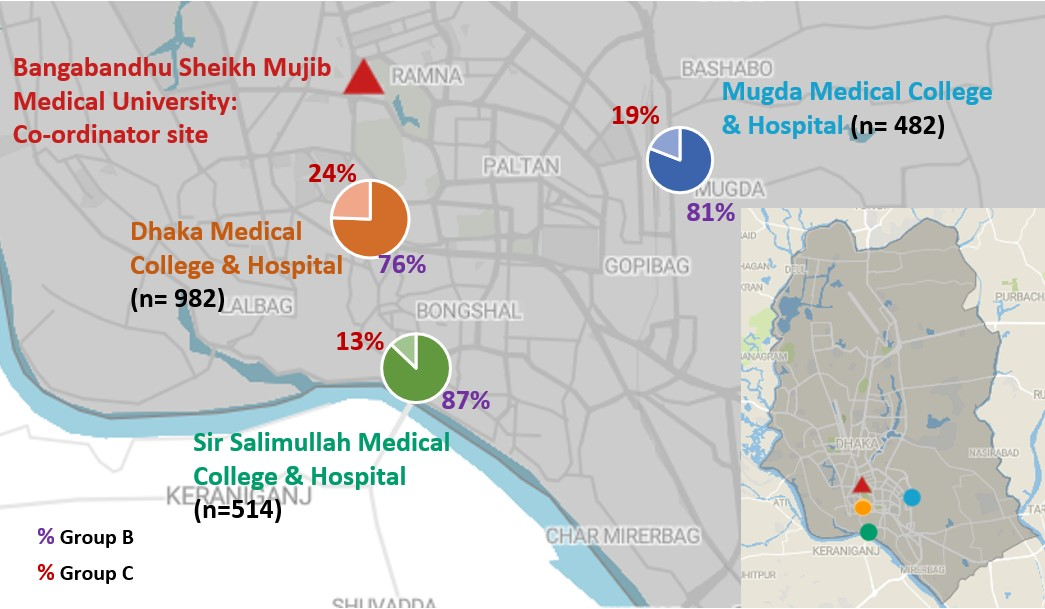

Supplement: S1 Fig — The red triangle represents the study co-ordinator site. The orange, blue and green circle depicts three data collection points. Site wise number of enrollment (black brackets) and percentage of group B and group C (purple and red respectively) are also shown. Map was plotted using Datawrapper website (available at https://www.datawrapper.de/_/2ew1H/). Basemap shapefile was extracted from OpenStreetMap with due permission from the concerned. (Open Database License, ODbL 1.0, see https://www.openstreetmap.org/copyright"). (TIF) [file pntd.0012686.s001.tif]

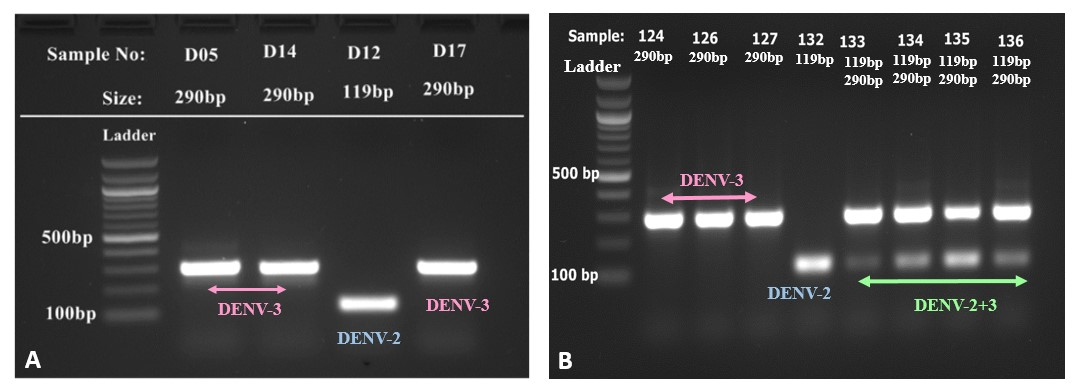

Supplement: S2 Fig — (S2A and S2B): Figure showing specimens from gel electrophoresis of DNA fragments generated at step 3. Fragment size of 290bp and 119bp suggests presence of DENV-3 and DENV-2 respectively. Concurrent infection by DENV-2 and DENV-3 can also be seen in S2B Fig. Presence of no other serotype was identified. (TIF) [file pntd.0012686.s002.tif]
