# Supplementary material for: Clinical and laboratory comparison of severe (Group B and C) Dengue cases with molecular characterization from 2019 epidemics in Dhaka, Bangladesh
Source: PLoS Negl Trop Dis. 2024 Nov 25;18(11):e0012686. doi: 10.1371/journal.pntd.0012686 (PMC11627391; doi:10.1371/journal.pntd.0012686)
Supplement: S1 Text — Table A: List of primers used in PCR reactions. Table B: Reaction condition for reverse transcriptase PCR. Table C: Reagent composition for reverse transcriptase PCR. Table D: Reaction condition of nested PCR. Table E: Reagent composition of Nested PCR. Table F: Serotypes and respective DNA fragment sizes. Table G: List of DENV-2 sequences used in the dendrogram. Table H: List of DENV-3 sequences used in the dendrograms. Table I: Serotype distribution between group B and C cases out of 81. Table J: Description of clinical presentations and laboratory parameters of 81 cases according to serotype distribution during the 2019 outbreak. Table K: Partial sequence of sample 1, 2, 3, 17 & 14. (DOCX) [file pntd.0012686.s003.docx]

Supplement of Methodology

S1.1 Data collection:

A semi-structured questionnaire was prepared in which clinical, epidemiological data, management, complications and outcome were recorded. Prior to recruitment diagnostic tests (dengue NS1 antigen, anti-dengue IgM and IgG RDT) were performed. Baseline tests (e.g.: CBC, renal function test, liver function test, serum electrolytes, urine RME, HbA1C etc.) and special laboratory tests (serum lipase, serum amylase, Chest X-ray, Ultrasound of whole abdomen etc.) were done as per indication and the attending physician's assessment.

S1.2 Sample collection, preparation and storage:

3mL venous blood was collected from each participant in red topped vacutainer for serotyping. The blood specimens were left at room temperature (25 to 30°C) for 30 minutes which facilitated their complete coagulation. Subsequently, they were centrifuged at 1,000 × g for 10 minutes at 4°C and stored at -20°C freezer before transported for serotyping to the laboratory of the Department of Genetic Engineering and Biotechnology, University of Dhaka, Bangladesh.

**S1.3 Dengue serotyping**

1. RNA extraction and Reverse transcriptase PCR:

RNA was isolated from 400 µL serum using NEB RNA extraction kit (Cat No: T2010S) according to manufacturer’s protocol and reverse transcriptase PCR was performed using Qiagen One step RT-PCR kit (Cat. No:210212). Primers used in this study were already designed and validated for dengue virus serotyping[2]. Primers along with their respective product sizes are listed in Table S1. In this step primer Dc2F and Dc2R were used to generate a 511 base pair long DNA fragment. As this primer pair binds to consensus regions of dengue genome that are similar among all serotypes, DNA fragment is generated from RNA of any dengue virus serotype. Reaction conditions and composition are listed in Table S2 and S3. Here reverse transcriptase produces cDNA from RNA genome and the cDNA later serves as template for replication by DNA polymerase.

**Table A: List of primers used in PCR reactions**

| **Primer** | **Sequence** | **Genome Position** | **Product size** |
| --- | --- | --- | --- |
| Dc2F | TCAATATGCTGAAACGCGCGAGAAACCG | 134-162 | 511 bp |
| Dc2R | TTGCACCAACAGTCAATGTCTTCAGGTTC | 616-644 |  |
| TS1 | TTGCACCAACAGTCAATGTCTTCAGGTTC | 568-586 | 482 bp |
| TS2 | CGCCACAAGGGCCATGAACAG | 232-252 | 119 bp |
| TS3 | TAACATCATCATGAGACAGAGC | 400-421 | 290 bp |
| TS4 | CTCTGTTGTCTTAAACAAGAGA | 506-527 | 392 bp |

**Table B: Reaction condition for reverse transcriptase PCR**

| Cycle Number | Steps | temp | time |
| --- | --- | --- | --- |
| 1 | cDNA synthesis | 55^0^ C | 30 min |
| 1 | Denaturation | 95^0^ C | 15 min |
| 40 | Denaturation | 95^0^ C | 30 sec |
|  | Annealing | 60^0^ C | 30 sec |
|  | Extension | 72^0^ C | 1 min |
| 1 | Final Extension | 72^0^ C | 10 min |

**Table C: Reagent composition for reverse transcriptase PCR**

| Reagents | Volume (µL) |
| --- | --- |
| water | 15 |
| 5X RT-PCR buffer | 5 |
| dNTP | 1 |
| 1 step Enzyme mix | 1 |
| Primer:  Dc2F, Dc2R | 0.5 for each |
| template | 2 |
| total | 25 |

1. **Nested PCR**

This step involves a subsequent run of polymerase chain reaction with a second set primers intended to amplify secondary target within the first run product of 511 bp length generated at step 2. This second round PCR generate DNA fragments of different sizes specific for each serotype. Primers Dc2F and TS1, TS2, TS3, TS4 were used during this reaction. While the Dc2F is common for all serotypes, the four reverse primers TS1, TS2, TS3 and TS4 are serotype specific and bind to serotype 1, 2, 3 and 4 respectively. Reaction condition and composition of reaction mix are listed in Table S4 and S5. Dream Taq DNA polymerase (Cat. No: EP0701) from thermo scientific and dNTP from NEB (Cat. No: N0447S) were used for PCR reaction. Instead of whole genome, using the pre-amplified 511bp segment as template directs the serotype specific primers to a distinct region and lowers the risk of miss priming and thereby increases specificity of the whole assay.

**Table D: Reaction condition of nested PCR**

| Cycle Number | Steps | temp | time |
| --- | --- | --- | --- |
| 1 | Denaturation | 95^0^ C | 5 min |
| 25 | Denaturation | 95^0^ C | 30 sec |
|  | Annealing | 50^0^ C | 1 min |
|  | Extension | 72^0^ C | 1 min |
| 1 | Final Extension | 72^0^ C | 5 min |

**Table E: Reagent composition of Nested PCR**

| Reagents | Volume (µL) |
| --- | --- |
| water | 17.375 |
| 10X reaction buffer | 2.5 |
| dNTP | 0.5 |
| Polymerase | 0.125 |
| Primers:  Dc2F, TS1, TS2, TS3, TS4 | 0.5 for each |
| Template | 2 |
| Total | 25 |

1. **Gel Electrophoresis:**

Step 3 generates DNA of different sizes based on the serotype of dengue virus present in the sample. The sizes of serotype specific products are given in Table S6. To understand the size of the DNA, the PCR products of step 3 are applied to gel electrophoresis. It involves running the DNA through agarose gel submerged in Tris acetate EDTA buffer by applying voltage difference. This technique seperates DNA based on their sizes and can be visualized under UV light as distinct band if the gel is stained with dyes like ethidium bromide. The size is estimated using comparing the band with ladder containing DNA of known sizes.

**Table F: Serotypes and respective DNA fragment sizes**

| **Serotype** | **Fragment size** |
| --- | --- |
| DEN-1 | 482 bp |
| DEN-2 | 119bp |
| DEN-3 | 290bp |
| DEN-4 | 392bp |

**S1.4 Sequences used in dendrogram**

**DENV-2:**

The dataset for DENV-2 consisted of already classified 40 sequences including 5 distinct genotypes as follows: Asian-I (n = 8), Asian-II (n = 5), Cosmopolitan (n = 23), American (n = 1), Asian/American (n = 2), Sylvatic (n=1). In addition, the sequence obtained in the present study was included along with 16 sequences that showed high levels of identity in BLAST search. Among the 16 sequences of BLAST search, 12 sequences were already reported to belong to cosmopolitan genotype. A total of 57 sequences were used for the phylogenetic analysis (Table S7).

**Table G: List of DENV-2 sequences used in the dendrogram**

| Bd2019 cD14A serotype2 50-440/1-390 |
| --- |
| MW512457.1 \|Dengue virus type 2\|DENV-2 cosmopolitan\|Singapore\|Singapore\|2016/212-601 |
| MW512473.1 \|Dengue virus type 2\|DENV-2 cosmopolitan\|Singapore\|Singapore\|2017/212-601 |
| OP684171.1 \|Dengue virus type 2\|Cosmopolitan\|China\|China\|2017-08-24/116-505 |
| LC436674.1 \|Dengue virus type 2\|cosmo\|Bangladesh\|Bangladesh\|2017-11/184-573 |
| LC436675.1 \|Dengue virus type 2\|cosmo\|Bangladesh\|Bangladesh\|2017-12/184-573 |
| MW512482.1 \|Dengue virus type 2\|DENV-2 cosmopolitan\|Singapore\|Singapore\|2018/212-601 |
| OP684182.1 \|Dengue virus type 2\|Cosmopolitan\|China\|China\|2018-08-01/187-576 |
| MN400316.1 \|Dengue virus type 2\|\|Bangladesh\|Bangladesh\|2018-09-15/26-415 |
| MN400313.1 \|Dengue virus type 2\|\|Bangladesh\|Bangladesh\|2018-10-06/20-409 |
| MW512498.1 \|Dengue virus type 2\|DENV-2 cosmopolitan\|Singapore\|Singapore\|2019/212-601 |
| ON908244.1 \|Dengue virus type 2\|\|China\|China\|2019-06-20/203-592 |
| MN328061.1 \|Dengue virus type 2\|\|Bangladesh\|Bangladesh\|2019-07-29/207-596 |
| MW599419.1 \|Dengue virus type 2\|\|Bangladesh\|Bangladesh\|2019-08-15/50-439 |
| MW720953.1 \|Dengue virus type 2\|\|China\|China\|2019-09/209-598 |
| OM791800.1 \|Dengue virus type 2\|Cosmopolitan\|Peru\|Peru\|2019-09-26/116-505 |
| OM791801.1 \|Dengue virus type 2\|Cosmopolitan\|Peru\|Peru\|2019-09-28/116-505 |
| LC410190.1 \|Dengue virus type 2 cosmo\|Thailand\|Thailand\|2016-10/184-573 |
| KX452030.1 \|Dengue virus type 2 cosmo\|Malaysia: Johor Bahru\|Malaysia\|2014-01/232-621 |
| KY427084.1 \|Dengue virus type 2 cosmo\|India: Thiruvananthapuram Kerala\|India\|2010-09-01/212-601 |
| KY921905.1 \|Dengue virus type 2 cosmo\|Singapore\|Singapore\|2015-03/212-601 |
| KX621247.1 \|Dengue virus type 2 cosmo\|China\|China\|2015/170-559 |
| KU517847.1 \|Dengue virus type 2 cosmo\|Philippines\|Philippines\|2015-08-03/212-601 |
| LC121816.1 \|Dengue virus type 2 cosmo\|\|\|2016-01/190-579 |
| KJ010186.1 \|Dengue virus type 2 cosmo\|Pakistan\|Pakistan\|2013-10-02/180-569 |
| JQ955624.1 \|Dengue virus type 2 cosmo\|India: Odisha\|India\|2011/212-601 |
| JX475906.1 \|Dengue virus type 2 cosmo\|India: Hyderabad\|India\|2009/212-601 |
| FJ196853.1 \|Dengue virus type 2 cosmo\|China: Guangzhou Guangdong Province\|China\|2003/212-601 |
| GQ398258.1 \|Dengue virus 2 cosmo\|Indonesia\|Indonesia\|1975/212-601 |
| GQ398259.1 \|Dengue virus 2\|cosmo\|Indonesia\|1976/212-601 |
| GQ398260.1 \|Dengue virus 2\|cosmo\|Indonesia\|1976/212-601 |
| GQ252677.1 \|Dengue virus type 2 cosmo\|Sri Lanka\|Sri Lanka\|2004/179-568 |
| FJ882602.1 \|Dengue virus type 2 cosmo\|Sri Lanka\|Sri Lanka\|1996/190-579 |
| FJ898454.1 \|Dengue virus type 2 cosmo\|India\|India\|2006/182-571 |
| EU056810.1 \|Dengue virus type 2 cosmo\|Burkina Faso\|Burkina Faso\|1983/212-601 |
| DQ645546.1 \|Dengue virus type 2 cosmo\|Taiwan\|Taiwan\|/189-578 |
| EU482672.1 \|Dengue virus type 2 cosmo\|Viet Nam: south\|Viet Nam\|2006/190-579 |
| AB189122.1 \|Dengue virus type 2 cosmo\|Indonesia: Sumatra\|Indonesia\|/212-601 |
| AY037116.1 \|Dengue virus 2 cosmo Australia: Townsville\|Australia\|/212-601 |
| AF359579.1 \|Dengue virus 2 cosmo\|\|\|/212-601 |
| JF730049.1 \|Dengue virus type 2 asian1\|Viet Nam: South\|Viet Nam\|2007/202-591 |
| GU131932.1 \|Dengue virus type 2 asian1\|Cambodia: KCH\|Cambodia\|2008/190-579 |
| GQ868542.1 \|Dengue virus type 2 asian1\|Thailand: Bangkok\|Thailand\|1994/190-579 |
| GQ868591.1 \|Dengue virus type 2 asian1\|Thailand\|Thailand\|1964/190-579 |
| FJ898452.1 \|Dengue virus type 2 asian1\|Thailand\|Thailand\|2003/190-579 |
| FJ639704.1 \|Dengue virus type 2 asian1\|Cambodia: KCH\|Cambodia\|2003/185-574 |
| EU482784.1 \|Dengue virus type 2 asian1\|Viet Nam: south\|Viet Nam\|2003/191-580 |
| DQ181798.1 \|Dengue virus type 2 asian1\|Thailand: Bangkok\|Thailand\|1999/212-601 |
| JF730050.1 \|Dengue virus type 2 asian2\|USA: California\|USA\|2007/210-599 |
| GQ398268.1 \|Dengue virus 2 asian2\|Indonesia\|Indonesia\|1975/212-601 |
| AF204177.1 \|Dengue virus type 2 asian2\|China:Hainan province\|China\|/212-601 |
| AF204178.1 \|Dengue virus type 2 asian2\|China:Guangxi province\|China\|/212-601 |
| AF038403.1 \|Dengue virus type 2 asian2\|\|\|/212-601 |
| KC294223.1 \|Dengue virus type 2 asian american\|Peru: Iquitos\|Peru\|2010-10-19/212-601 |
| HQ999999.1 \|Dengue virus type 2 asian american\|Guatemala: Guatemala City\|Guatemala\|2009/212-601 |
| GQ868592.1 \|Dengue virus type 2 american\|Colombia\|Colombia\|1986/190-579 |
| EF105381.1 \|Dengue virus type 2 sylvatic\|Cote dIvoire\|Cote dIvoire\|1980/212-601 |

**DENV-3:**

The dataset for DENV-3 consisted of 46 sequences belonging to 4 distinct genotypes as follows: I (n = 10), II (n = 14), III (n = 17), and V (n = 5). In addition, three of five sequences obtained in the present study were included. Two sequences of this study were not included as large portion of their ends had to be trimmed due to low-quality reads. An additional 13 sequences that showed high levels of identity to the sequences obtained in the present study were also included. A total of 62 sequences were used for the phylogenetic analysis. Isolates of genotype IV could not be included due to lack of C-prM sequences of this genotype (Table S8).

**Table H: List of DENV-3 sequences used in the dendrograms**

| Bd2019 cD1A serotype3 50-400 |
| --- |
| Bd2019 cD2A serotype3 50-400 |
| Bd2019 cD17A serotype3 60-400 |
| MW599415.1 2019 rangpur Dengue virus 3 isolate Rangpur01 polyprotein (POLY) gene partial cds |
| MW599418.1 2019 dhaka Dengue virus 3 isolate Dhaka01 polyprotein (POLY) gene partial cds |
| lcl\|Query 40186:24-375 MN400330 \|Bangladesh\|2018/09/18\| |
| lcl\|Query 40213:27-378 MN400331 \|Bangladesh\|2018/10/26\| |
| lcl\|Query 40212:24-375 MN400333 \|Bangladesh\|2018/09/16\| |
| 191-542 LC436677.1 Dengue virus 3 2017 Dhaka B17-1479 RNA nearly complete genome |
| 197-548 MN922035 \|China\|2019/06/23\| |
| 200-551 MN922033.1 2019 china Dengue virus 3 |
| lcl\|Query 40187:211-562 MN018388 \|China\|2017/11/07\| |
| lcl\|Query 40234:66-417 MG182045 \|Malaysia\|2016/08/15\| |
| lcl\|Query 40274:211-562 MN018383.1\|Indonesia\|2014\| Dengue virus 3 isolate D14014 complete genome |
| lcl\|Query 40129:6-357 MH612618 \|Thailand\|2017/06/17\| |
| lcl\|Query 40108:117-468 MN083246 \|Sri Lanka\|2017/11\| |
| OP410997.1:1-900 Dengue virus 3 3 2019 singapore complete genome |
| OP410998.1:1-900 Dengue virus 3 32018 singapore complete genome |
| lcl\|Query 40107:55-405 DENV3 3 MK517808 \|India\|2015/09\| |
| lcl\|Query 40101:67-417 DENV3 3 MK829123 \|India\|2018/10\| |
| lcl\|Query 40143:1-227 MT012479 \|Sudan\|2019/10/15\| |
| KY921906.1 \|Dengue virus type 3 1\|Singapore\|Singapore\|2015-06 |
| KC762691.1 \|Dengue virus 3 1\|Indonesia: Makassar\|Indonesia\|2008-04-10 |
| EU081223.1 \|Dengue virus type 3 1\|Singapore\|Singapore\|2005 |
| JN406515.1 \|Dengue virus type 3 1\|Australia\|Australia\|2008 |
| KY794787.1 \|Dengue virus type 3 1\|Papua New Guinea: Madang\|Papua New Guinea\|2007-10-09 |
| KX380839.1 \|Dengue virus type 3 1\|Singapore\|Singapore\|2012 |
| JQ920486.1 \|Dengue virus type 3 1\|New Caledonia\|New Caledonia\|1996-11-18 |
| EF629370.1 \|Dengue virus type 3 5\|Brazil\|Brazil\| |
| KU050695.1 \|Dengue virus type 3 5\|Philippines\|Philippines\|1956 |
| JQ920480.1 \|Dengue virus type 3 1\|French Polynesia: Tahiti\|French Polynesia\|1996-09-04 |
| AF317645.1 \|Dengue virus type 3 5\|China: Guangxi\|China\| |
| JN697379.1 \|Dengue virus type 3 5\|Brazil\|Brazil\|2006 |
| KM190937.1 \|Dengue virus type 3 5\|Philippines\|Philippines\|1964 |
| KC762686.1 \|Dengue virus 3 1\|Indonesia: Makassar\|Indonesia\|2007-07-02 |
| FJ882576.1 \|Dengue virus type 3 3\|Nicaragua\|Nicaragua\|1994 |
| JX669490.1 \|Dengue virus type 3 3\|Brazil: Pernambuco\|Brazil\|2002 |
| NC 001475.2 \|Dengue virus type 3 3\|Sri Lanka\|Sri Lanka\| |
| DQ675531.1 \|Dengue virus type 3 2\|Taiwan\|Taiwan\|1998 |
| GQ868593.1 \|Dengue virus type 3 2\|Thailand\|Thailand\|1973 |
| KF955477.1 \|Dengue virus type 3 2\|India\|India\|1984 |
| AY496877.2 \|Dengue virus type 3 2\|Bangladesh\|Bangladesh\|2002 |
| AY496874.2 \|Dengue virus type 3 2\|Bangladesh\|Bangladesh\|2002 |
| AY496873.2 \|Dengue virus type 3 2\|Bangladesh\|Bangladesh\|2002 |
| AY496871.2 \|Dengue virus type 3 2\|Bangladesh\|Bangladesh\|2002 |
| KU509280.1 \|Dengue virus type 3 2\|Thailand: Ko Samui\|Thailand\|2011 |
| GQ199887.1 \|Dengue virus type 3 3\|Sri Lanka: Western Province\|Sri Lanka\|1983 |
| FJ687448.1 \|Dengue virus type 3 2\|Thailand: Kampheange Phet\|Thailand\|2001 |
| KF955460.1 \|Dengue virus type 3 2\|Viet Nam\|Viet Nam\|2008 |
| mawahib 59269:212-562 AY679147.1 \|Dengue virus type 3 3\|Brazil\|Brazil\| |
| mawahib 59267:176-526 FJ182013.1 \|Dengue virus type 3 3\|USA: Puerto Rico\|USA\|1998 |
| mawahib 59266:191-541 FJ882575.1 \|Dengue virus type 3 3\|Mozambique\|Mozambique\|1985 |
| mawahib 59258:212-562 KJ737429.1 \|Dengue virus type 3 2\|Thailand\|Thailand\|1994 |
| mawahib 59261:191-541 EU482452.1 \|Dengue virus type 3 2\|Viet Nam: south\|Viet Nam\|2006 |
| mawahib 59259:212-555 KJ622197.1 \|Dengue virus type 3 2\|China\|China\|2013 |
| mawahib 59260:211-555 KF824903.1 \|Dengue virus type 3 2\|China\|China\|2013 |
| mawahib 59268:212-562 AY770511.2 \|Dengue virus type 3 3\|India: Gwalior\|India\| |
| mawahib 59265:212-562 JF504679.1 \|Dengue virus type 3 3\|China: Zhejiang Yiwu\|China\|2009-09 |
| mawahib 59262:13-363 MH623006.1 \|Dengue virus type 3 3\|India\|India\|2016 |
| mawahib 59263:26-376 MK204364.1 \|Dengue virus type 3 3\|Saudi Arabia\|Saudi Arabia\|2016 |
| mawahib 59264:73-423 MG973738.1 \|Dengue virus type 3 3\|India\|India\|2017 |
| KU509279.1 \|Dengue virus type 3 1\|Philippines\|Philippines\|2008 |

Supplement of Result:

**S2.1 Dengue serotype and case profile**

**Table I: Serotype distribution between group B and C cases out of 81 (p= ns)**

| **Serotype detected**  **n(%)** | Total  **(n=81)** | **Group B**  **(n=53)** | **Group C**  **`(n=28)** |
| --- | --- | --- | --- |
| DEN-2 | 6 (7.4) | 3 (50) | 3(50) |
| DEN-3 | 64 (79) | 43 (67.2) | 21 (32.8) |
| DEN- 2 and 3 | 11 (13.6) | 7 (63.6) | 4 (36.4) |

**Table J: Description of clinical presentations and laboratory parameters of 81 cases according to serotype distribution during the 2019 outbreak**

| **Characteristics** | **DENV-2**  **(n=6)** | **DENV-3**  **(n-64)** | **DENV- 2+3**  **(n=11)** | **p-value ^*^** |
| --- | --- | --- | --- | --- |
| Age (median, IQR) | 22,20- 36 | 26,17- 36 | 25,23-35 | 0.7915 |
| Duration of fever(median, IQR) | 5.5,5- 7 | 5,4- 6 | 5,4- 6 | 0.4485 |
| Headache, n (%) | 4 (66.7) | 47 (73.44) | 9 (81.82) | 0.7678 |
| Joint pain, n (%) | 3(50) | 31 (48.44) | 9 (81.82) | 0.1210 |
| Retro orbital pain, n (%) | 2 (33.33) | 25 (39.06) | 7 (63.64) | 0.2828 |
| Rash, n (%) | 2 (33.33) | 22 (34.38) | 8 (72.73) | 0.0529 |
| Myalgia, n (%) | 3 (50) | 41 (64.06) | 10 (90.91) | 0.1456 |
| Lethargy, n (%) | 1 (16.67) | 39 (60.94) | 9 (81.82) | **0.0314** |
| GI symptoms | 5 (83.3) | 59 (92.2) | 11 (100) | 0.0969 |
| Anorexia | 3 (50) | 48 (75) | 10(91) |  |
| Nausea | 1 (16.7) | 21 (32.8) | 5 (45.4) |  |
| Vomiting | 4 (66.7) | 49 (76.5) | 8 (72.7) |  |
| Diarrhea | 2 (33.3) | 35 (54.7) | 3 (27.3) |  |
| Abdominal pain | 2 (33.3) | 20 (31.2) | 1 (9) |  |
| Bleeding manifestations | 4 (66.7) | 33 (51.6) | 8 (72.73) | 0.3630 |
| **Melaena** | 4 (66.7) | 24 (37.5) | 7 (63.6) |  |
| **Hematochezia** | 1 (16.7) | 4 (6.2) | 0 |  |
| **Epistaxis** | 0 | 2 (3.13) | 0 |  |
| **Haematemesis** | 0 | 2 (3.13) | 2 (18.2) |  |
| **Hemoptysis** | 0 | 2 (3.13) | 0 |  |
| **Hematuria** | 0 | 0 | 1 (9.1) |  |
| **Gum bleed** | 0 | 3 (4.7) | 0 |  |
| **PV bleed** | 0 | 2 (3.13) | 1 (9.1) |  |
| **Bleed from IV puncture site** | 0 | 1 (1.56) | 0 |  |
| Primary infection** | 6 (100%) | 59 (92.2%) | 11 (100%) | 0.4928 |
| Hemoglobin (g/dl) | 12.3,12.2- 12.8 | 13,11.8- 14.4 | 13.8,13- 15 | 0.1072 |
| Hematocrit (%) | 38,36.5- 39 | 39,35.4- 42.3 | 41.5,37.5- 45.2 | 0.3208 |
| Total WBC count (/µL) | 3440,2920- 7357.5 | 5180,3750- 6525 | 4300,3600- 5850 | 0.7315 |
| N:L ratio | 1.8,1.6- 2 | 2.5,1.6- 3.3 | 2.1,1.2- 5.4 | 0.7190 |
| Platelet count(/L) | 125500,60000- 134000 | 110000,49500- 166000 | 105000,45500- 162500 | 0.8514 |

*****Mann-Whitney and Chi-square test (two-sided) was applied to identify the level of significance. **Primary infection was concluded by NS1 antigen RDT positivity. P-value <0.05 was considered statistically significant (bold).

**S2.2 Sequence analysis and phylogenetic relationship**

| **Table K: Partial sequence of sample 1, 2, 3, 17 & 14** | | |  |
| --- | --- | --- | --- |
| Sample | Serotype | Sequence | GenBank  Accession  Number |
| 1 | 3 | >Bd2019_cD1A_serotype3_50-400  TGGCCAAGGACCAATGAAATTGGTTATGGCGTTCATAGCTTTCCTCAGATTTCTAGCCATTCCACCGACAGCGGGAATCTTGGCTAGATGGGGAACCTTCAAGAAGTCGGGGGCTATTAAGGTCCTGAGAGGCTTCAAGAGGGAGATCTCAAATATGCTGAGCATTATCAACAGACGGAAAAAGACATCGTTCTGTCTCATGATGATGTTACCAGCAACACTTGCTTTCCACTTGACTTCACGAGATGGAGAGCCGCGCATAATTGTGGGGAAGAATGAAAGAGGAAAATCCCTACTTTTTAAGACAGCCTCTGGAATCAACATGTGCACACTCATAGCCATGGATCTGGGA | [OR726315](https://www.ncbi.nlm.nih.gov/nuccore/OR726315) |
| 2 | 3 | > Bd2019_cD2A_50-400  TGGCCAAGGACCAATGAAATTGGTTATGGCGTTCATAGCTTTCCTCAGATTTCTAGCCATTCCACCGACAGCGGGAATCTTGGCTAGATGGGGAACCTTCAAGAAGTCGGGGGCTATTAAGGTCCTGAGAGGCTTCAAGAGGGAGATCTCAAATATGCTGAGCATTATCAACAGACGAAAAAAGACATCACTCTGTCTCATGATGATGTTACCAGCAACACTTGCTTTCCACTTGACTTCACGAGATGGAGAGCCGCGCATGATTGTGGGGAAGAATGAAAGAGGAAAATCCCTACTTTTTAAGACAGCCTCTGGAATCAACATGTGCACACTCATAGCCATGGATCTGGGAG | [OR726316](https://www.ncbi.nlm.nih.gov/nuccore/OR726316) |
| 3 | 3 | > Bd2019_cD3A_ serotype3-50-309  TGGCCAAGGACCAATGAAATTGGTTATGGCGTTCATAGCTTTCCTCAGATTTCTAGCCATTCCACCGACAGCGGGAATCTTGGCTAGATGGGGAACCTTCAAGAAGTCGGGGGCTATTAAGGTCCTGAGAGGCTTCAAGAGGGAGATCTCAAATATGCTGAGCATAATCAACAGACGGAAAAAGACATCACTCTGTCTCATGATGATGTTACCAGCAACACTTGCTTTCCACTTGACTTCACGAGATGGAGAGCCGCGCA | [OR726317](https://www.ncbi.nlm.nih.gov/nuccore/OR726317) |
| 17 | 3 | > Bd2019_cD17A_ serotype3_60-400  CAATGAAATCGGTTATGGCGTTCATAGCTTTCCTCAGATTTCTAGCCATTCCACCGACAGCGGGAATCTTGGCTAGATGGGGAACCTTCAAGAAGTCGGGGGCTATTAAGGTCCTGAGAGGCTTCAAGAGGGAGATCTCAAATATGCTGAGCATTATCAACAGACGAAAAAAGACATCACTCTGTCTCATGATGATGTTACCAGCAACACTTGCTTTCCACTTGACTTCACGAGATGGAGAGCCGCGCATGATTGTGGGGAAGAATGAAAGAGGAAAATCCCTACTTTTTAAGACAGCCTCTGGAATCAACATGTGCACACTCATAGCCATGGATCTGGGA | [OR726318](https://www.ncbi.nlm.nih.gov/nuccore/OR726318) |
| 14 | 2 | > Bd2019_cD14A_ serotype2_50-440  AGGGACGAGGACCACTGAAACTGTTCATGGCCCTGGTGGCATTCCTTCGTTTCCTAACAATCCCGCCAACAGCAGGGATATTAAAAAGATGGGGAACAATCAAAAAATCAAAGGCTATCAATGTCTTGAGAGGGTTTAGGAAAGAGATTGGAAGGATGCTGAACATCTTGAACAGGAGACGCAGAACTGCAGGTATAATTATTATGATGATCCCAACAGTGATGGCGTTCCATTTAACCACACGCAATGGAGAACCACACATGATCGTCAGTAGACAAGAGAAAGGGAAAAGTCTTCTGTTTAAAACAGAGAACGGTGTGAACATGTGCACCCTCATGGCCATGGATCTTGGTGAACTGTGTGAAGACACAATCACTTATAATTGTCCTC | [OR726319](https://www.ncbi.nlm.nih.gov/nuccore/OR726319) |

**References:**

1. Revised guideline for clinical management of dengue. [cited 1 Aug 2021]. Available: https://dghs.gov.bd/index.php/en/home/5431-revised-guideline-for-clinical-management-of-dengue

2. Lanciotti RS, Calisher CH, Gubler DJ, Chang GJ, Vorndam A V. Rapid detection and typing of dengue viruses from clinical samples by using reverse transcriptase-polymerase chain reaction. J Clin Microbiol. 1992;30: 545–551. doi:10.1128/JCM.30.3.545-551.1992
