# Supplementary material for: Clinical and laboratory comparison of severe (Group B and C) Dengue cases with molecular characterization from 2019 epidemics in Dhaka, Bangladesh
Source: PLoS Negl Trop Dis. 2024 Nov 25;18(11):e0012686. doi: 10.1371/journal.pntd.0012686 (PMC11627391; doi:10.1371/journal.pntd.0012686)
Supplement: Attachment — Submitted filename: PNTD-D-23-01524_Reviewer Comments.docx [file pntd.0012686.s004.docx]

**Reviewer’s Comment**

**PLOS Neglected Tropical Diseases**

**Project Title:** Clinical and laboratory comparison of severe (Group B and C) Dengue cases with molecular characterization from 2019 epidemics in Dhaka, Bangladesh

**Manuscript Number:** PNTD-D-23-01524

**Abstract**

1. Sentence: “The study enrolled 1978 participants who were grouped into group B and C according to national guidelines”.

Issue – Define what is meant by group B and C

2. Sentence: “Median (IQR) of the cohort was 26(11 to 41)”

Issue – median of what? Besides, it seems it is a repetition.

3. Sentence: “Only 5.56% were secondary infections”

Issue: Round up 5.56% to one decimal point as precision measurement is not required here.

4. Sentence: “Only 13 (0.66%) patients succumbed to death …..”

Issue: State the number of patients without qualifying as ‘only’ and round up all decimals to one decimal point across the entire manuscript.

5. Sentence: “………. 12 of them belonged to group B.”

Issue: the sentences that follow should describe the characteristics of the group B who had more deaths.

6. Sentence: “Out of 81 samples 79% patients were serotyped as DENV-3, followed by

DENV 2,3 (13.6% ) and DENV-2 (7.4%).”

Issue: Do you mean, ‘Out of 81 samples **collected from patients**, 79% were …..”? Also, it seems DENV 2 is being reported twice. Please clarify

**Methods**

**Study site and enrollment:** It was mentioned that the study was done from 1st July to 31st December, 2019 whereas, in the author summary and abstract sections, study period was reported as August to December 2019? Please harmonize.

**Results**

**Dengue serotyping:** “the former two serotypes”. It is advisable to mention the “former serotypes” since not all readers are familiar with the former serotypes.

- Recast this sentence for clarity: “Among the three patterns of identified serotypes majority

(43, 67.2%) of the cases belonged to group B in DENV-3 and DENV-2 and 3 co infection (7, 63.6%).”

**Discussion**

- It will be helpful to define ‘primary infection’ in the manuscript.
- “ICU transfers and mortality rate was also minimal than other sites [44].” Expatiate on what is meant by ‘other sites’

**Recommendation**: I will suggest that the manuscript is reviewed by an English editor.
