# Supplementary material for: Clinical and laboratory comparison of severe (Group B and C) Dengue cases with molecular characterization from 2019 epidemics in Dhaka, Bangladesh
Source: PLoS Negl Trop Dis. 2024 Nov 25;18(11):e0012686. doi: 10.1371/journal.pntd.0012686 (PMC11627391; doi:10.1371/journal.pntd.0012686)
Supplement: Attachment — Submitted filename: review report PNTD.docx [file pntd.0012686.s005.docx]

**Clinical and laboratory comparison of severe (Group B and C) Dengue cases with molecular characterization from 2019 epidemics in Dhaka, Bangladesh**

**General comments**

- Overall, the study in its current form is unacceptable. Language, grammar, and writing style need some serious improvements. Practically, authors need to rewrite the whole manuscript before resubmitting.
- Much of the methodology and results are given in the supplement files which need to be incorporated into the main manuscript file because it is very inconvenient for the reader to keep switching between the files. Summarize the methodology and cite references where required to save space.

**Methodology**

- Ethical considerations should be the first paragraph of the methodology.
- A map of the country/city pointing out the location of the study sites might be included. Please give information about the study site as well.
- **Study site and enrollment:** Briefly explain the criteria for the classification of dengue patients into suspected, probable, and confirmed cases. Although reference has been provided but brief description of the criteria will facilitate the reader.
- Why patients younger than 16 years were excluded from the study? It is an important cohort, and their data could improve the study quality.
- Since dengue has been circulating in Bangladesh for many years, it is possible that many cases were both IgM and IgG positive. They could have been included in the study as well.

**Results**

- Figure 1: Refer to the figure in the text as well.
- Please compare clinical presentation with similar symptoms among both groups and then compare hemorrhagic manifestations.
- **Demographic characteristics:** Firstly, discuss general characteristics (like age, gender, etc.). Afterward, characters of different groups and sub-groups might be discussed.
- Much data is given in the supplement files, however, baseline data related to demographic profile, and clinical presentation might be given in the main manuscript file. It would be very difficult for the reader to keep on switching between the files.
- It has been mentioned that many of the patients from group C were reported outside of Dhaka, however, nothing has been mentioned about how many were reported outside Dhaka in group B
- What is meant by service holders? Is it a specific designation in Bangladesh?
- Throughout the manuscript, IQR has been mentioned as ±N which is not an IQR.
- Whether the com-morbidities were diagnosed or self-reported by the patients? If self-reported by the patients, what about those comorbidities not reported by the patients?
- A many places in results, S is written as short for serum which is a non-standard abbreviation. Please write “serum” instead of “S”.
- Any patient with bacterial infection was excluded from the study so why antibiotic was given to one patient?
- DENV Serotyping: It is not clear which former infection authors are talking about. Is it "concurrent infections"?
- No tests were performed by the authors to confirm primary or secondary infections. Therefore, the authors cannot claim that all infections were primary infections.
- Genotyping: Please submit the sequence to Genbank to generate accession numbers which could be mentioned in the manuscript along with percentage similarity.

**Discussion**

- Please follow a logical order to write results and discussion, starting from the demographic characteristics, followed by clinical signs and symptoms, and finally DENV serotypes and Genotypes.
- Write dengue virus serotypes as DENV-1, DENV-2, DENV-3, and DENV-4 throughout the manuscript.

**Table 1**

Please avoid non-standard abbreviations like “S. electrolyte”. Write full “Serum”. Correct wherever required.

IQR or interquartile range is a range, therefore, not a single value.

- Why median values instead of mean values were calculated? Please calculate mean values for each variable to make them more comparable with other studies.
- Table 1 should be about the baseline characters of the study population. Signs and symptoms could also be incorporated in Table 1.
- Label "total" instead of "all"
- Where chi-square was applied as all the variables were quantitative?

**Table 3**

- Please mention only significant signs and symptoms in the text. There is no need to make a table for it.
- Please also discuss which hemorrhagic manifestation and GI manifestations were significantly different in these groups.

**Supplement file**

- S.1.1 Overall English needs to be improved. Nonstandard abbreviations like S. for serum must be avoided. Please write in full wherever required.
- Replace “Baseline tests (e.g. CBC, )

Table S10, S11: Characteristics column is not mentioned as n, %. Please correct.

Write “P-value” as “p-value” throughout the manuscript

The symbols used for footnotes in this study are non-standard. Please refer to the journal guidelines.

Table S10: Please write the duration of fever as mean ± SD

Age and gender distribution should be part of the demographic profile rather than the clinical presentation table.

How primary and secondary infections were differentiated, based upon any test or self-reported by the patients. Self-reported data cannot be relied upon as most of the dengue infections remain subclinical or asymptomatic.

Table S11: What is meant by TT

There is no need to write vitals if they remain normal. Write only if there was an increase or decrease due to dengue fever, excluding all other confounding factors. Mention how many patients' vitals were deranged.

S 2.2. There is no need to give photos of all the gels. Please give one or two properly labeled gel diagram(s) showing different sizes of bands and DNA ladder.

Figure S2.2 c and H are not available

**References**

- References 1 and 14 are the same
