# Supplementary material for: Clinical and laboratory comparison of severe (Group B and C) Dengue cases with molecular characterization from 2019 epidemics in Dhaka, Bangladesh
Source: PLoS Negl Trop Dis. 2024 Nov 25;18(11):e0012686. doi: 10.1371/journal.pntd.0012686 (PMC11627391; doi:10.1371/journal.pntd.0012686)
Supplement: Attachment — Submitted filename: Response to reviewers.docx [file pntd.0012686.s006.docx]

**Response to reviewers**

**PLOS Neglected Tropical Diseases**

**Project Title:** Clinical and laboratory comparison of severe (Group B and C) Dengue cases with molecular characterization from 2019 epidemics in Dhaka, Bangladesh

**Manuscript Number:** PNTD-D-23-01524

**Reviewer's Responses to Questions/ comments (reviewer 1)**

*Reviewer #1:* The project titled "Clinical and laboratory comparison of severe (Group B and C) Dengue cases with molecular characterization from 2019 epidemics in Dhaka, Bangladesh" had very clear objectives to describe the clinical and laboratory variations among severe dengue cases. The authors also conducted a serotype survey to enable them predict future changes in the clinical or serological profile of this virus. The population studied was from a dengue fever outbreak that happened in 2019. The sample size was sufficient. The clinical and laboratory evaluations were appropriate.
*Reviewer #1*: The results were clearly presented and they did match the analysis plan.  The quality of the figures are good and clear.

*Reviewer #1*: The authors concluded that emergence of novel strains and co-circulation of several strains are a significant problem in Bangladesh which could predispose to more severity of cases in the future. The limitation of serotyping all samples due to limited resources was mentioned. The public health importance of the study was mentioned.

*Reviewer #1:* The study described the changing clinical presentation of dengue cases which is very relevant for the global community because of the changing dynamics of vector borne diseases particularly in this era of climate change

*Response: Thank you for your observations and kind appreciation. We appreciate you taking your time to review the manuscript. We have reformed the manuscript as per requirement.*

*Reviewer #1: Other observations-*

**Abstract**

1. Sentence: “The study enrolled 1978 participants who were grouped into group B and C according to national guidelines”.

Issue – Define what is meant by group B and C

*Response: Thank you for your observation. It was previously described only in the main manuscript as there’s a word limit to abstract. We have added the definition as per comment, which now reads as*

*“ The study enrolled 1978 participants who were grouped into group B (Patients with warning signs*^1^ *or risk factors who should be admitted for close observation as they approach critical phase) and C ( Patients having severe plasma leakage leading to dengue shock and/or fluid accumulation with respiratory distress, severe organ impairment and severe metabolic abnormalities ) according to national guidelines.”*

*[Page 02; Line 35-40]*

2. Sentence: “Median (IQR) of the cohort was 26(11 to 41)”

Issue – median of what? Besides, it seems it is a repetition.

*Response: Thank you for your observation. The redundant part omitted.*

3. Sentence: “Only 5.56% were secondary infections”

Issue: Round up 5.56% to one decimal point as precision measurement is not required here.

*Response: Done.*

*[Page 3; Line: 52]*

4. Sentence: “Only 13 (0.66%) patients succumbed to death …..”

Issue: State the number of patients without qualifying as ‘only’ and round up all decimals to **one decimal point across the entire manuscript**

*Response: Done.*

*[Page 3; Line: 50]*

5. Sentence: “………. 12 of them belonged to group B.”

Issue: the sentences that follow should describe the characteristics of the group B who had more deaths.

*Response: Thank you for the comment. Sentence rephrased. It is now reads as ‘ 12 of them belonged to group B who mostly presented with GI manifestations (99.9%) and melaena (12%)’*

*[Page 3; Line: 50-52]*

6. Sentence: “Out of 81 samples 79% patients were serotyped as DENV-3, followed by

DENV 2, 3 (13.6%) and DENV-2 (7.4%).”

Issue: Do you mean, ‘Out of 81 samples collected from patients, 79% were …..”? Also, it seems DENV 2 is being reported twice. Please clarify

*Response: Thank you for your observation. We serotyped 81 samples from enrolled cases who were NS1 positive. Out of which DENV-3, DENV-2 and co infection with DENV-2 and 3 were detected in 64 (79%), 6 (7.4%) and 11 (13.58%) samples respectively. The sentence changed for clarification as,*

*“Furthermore, 81 samples from enrolled participants were serotyped and majority (79%) yielded DENV-3, followed by DENV 2 & 3 co-infection (13.6%) and DENV-2 alone (7.4%).”*

*[Page 03; Line: 58-60]*

**Methods**

**Study site and enrollment:** It was mentioned that the study was done from 1st July to 31st December 2019 whereas, in the author summary and abstract sections, study period was reported as August to December 2019? Please harmonize.

*Response: Thank you for your observation. We have corrected it to “1^st^ July to December 31^st^ 2019”.*

*[Page 07; Line: 129]*

**Results**

**Dengue serotyping:** “the former two serotypes”. It is advisable to mention the “former serotypes” since not all readers are familiar with the former serotypes.

*Response: Thank you for your observation. Rephrased for clarity as, “Among 86 extracted RNA samples 81 was successfully serotyped out of which DENV-3, DENV-2 and co infection with DENV-2 and 3 were detected in 64 (79 %), 6 (7.4%) and 11 (13.58%) samples respectively”*

*[Page 20; Line: 276- 278]*

- Recast this sentence for clarity: “Among the three patterns of identified serotypes majority (43, 67.2%) of the cases belonged to group B in DENV-3 and DENV-2 and 3 co infection (7, 63.6%).”

*Response: Thank you for your observation. Rephrased as, “Patients who were infected with DENV-3 or had co-infection (DENV-2 &3) mostly developed group B infection (>60%)”.*

*[Page 20; Line: 280- 282]*

**Discussion**

- It will be helpful to define ‘primary infection’ in the manuscript.

*Response: Thank you for your observation. Added in methodology which reads, “Primary infection were reported by laboratory evidence of NS1 or Anti dengue IgM”*

*[Page 08; Line: 147]*

- “ICU transfers and mortality rate was also minimal than other sites [44].” Expatiate on what is meant by ‘other sites’`

*Response: Thank you for your observation. Corrected as “ICU transfers and mortality rate was also minimal than other hospital reports of the same outbreak* ^2^*.”*

*[Page 29; Line: 434-435]*

**Reviewer's Responses to Questions/ comments (reviewer 2)**

Reviewer #2: How were the cases classified as primary or secondary infection. Details not provided.

*Response: Thank you for your comment. We have added the required part in the methodology as,*

*“Primary infection were marked by laboratory evidence of NS1 or Anti dengue IgM and secondary infections were declared when patients had Anti dengue IgG positivity. “*

*[Page 08; Line: 147-149]*

Reviewer #2: in Table 2 fluid requirement was calculated according to what criteria? Also is this requirement per 24 hours?

*Response: Thanks for pointing this out. Fluid requirement was individualized in every patient according to national guidelines. Which depends on patient’s symptoms and signs, body weight and their subsequent follow up. The patients were managed by the ward physicians who had no role in the study. The study physicians collected the information from patient and patient’s hospital log at one point of time.*

*The table shows the total requirement of patients in hospital admission time after enrollment.*

*N.B: The old table 2 is now table 4 as per new changes.*
In types of fluid only 84.9 % and 88% in group B and group C received fluid and 15 % didnt receive any crystalloid which is the first fluid given in patients with preshock or shock. Kindly explain this.

*Response: Thank you for your observation. 84.9% of group B patients received IV crystalloids. As the table highlights only IV fluids this data is not representative of total fluid intake of a patient. The other 15% of group B patients did not need IV fluids and were managed with oral fluid and oral rehydration solution.*

*88.4% of group C patients received crystalloids and the rest were managed with colloids, both crystalloids and colloid and, blood transfusion.*

Also 49 patients received platelet transfusion more in group B rather than group C which showed more hemorrhagic manifestations as quoted in table 3.

*Response: Thank you for your comment.*

*49 patient received platelet transfusion out of total cohort (old table 2, now table number changed to 4)*

*In old version of this manuscript Table 3 (before correction) showed the Description of clinical presentations and laboratory parameters of 81 cases* ***according to serotype*** *distribution during the 2019 outbreak stratified as DENV-2, DENV-3 and DENV-2 and 3 co infection(now shifted to supplement table -10 page 14, as per new correction).*

*But you have correctly observed the fact that the bleeding manifestations were more frequently seen in group C (42%) than in group B (37.2%) [Shown in new table 2 (old supplement table 10)]. Although the differences were not statistically significant (p=0.08).*

*According to our national guideline, we seldom use platelet transfusions in these cases except for some special situation. Fresh blood transfusion still remains the first line therapy in these cases, and you can observe it in table 4 that group C patients got more blood transfusions (5.5 vs 5.2). Again, these are differences were not significant statistically as mentioned in table 4.*

*The guideline for clinical management of dengue in Bangladesh can be downloaded from the below link*

*https://old.dghs.gov.bd/index.php/en/home/5431-revised-guideline-for-clinical-management-of-dengue*

Reviewer #3: (No Response)

**Additional comments:**
1.      **Copy editing is needed**

*Response: Thanks. Edited as much as possible.*
2.      Abstract, “forecast future changes in the clinical or serological profile of this virus”. While I agree that the study may help future planning, the study analysis did not forecast changes and it does not appear to be a clear study purpose. Please strengthen the relevant analysis and discussion, or alternatively remove in the abstract and revise accordingly in the main text,.

*Response: Thank you for your observation. Sentence rewritten as, “with an aim to describe the clinical and laboratory variations among severe dengue cases and to conduct a serotype survey. This might help to understand the future changes in the clinical or serological profile of this virus.”*

*[Page 2; Line: 32-35]*

3.      Tables 1-3. Please use standard symbols for the footnotes

*Response: Done.*
4.      Tables 1-3. Please state the meaning of the bolded numbers (p-values and numbers/%)

*Response: Done.*
5.      Table 2. Please remove the level of significance in the footnote.

*Response: Done.*
6.      Table 1 & 3. Please provide the units directly in the table.

*Response: Done.*

**General comments**

- Overall, the study in its current form is unacceptable. Language, grammar, and writing style need some serious improvements. Practically, authors need to rewrite the whole manuscript before resubmitting.

*Response: We have not heard anything re this from reviewer 1 and 2. However, we have checked the grammar and language and improved accordingly.*

- Much of the methodology and results are given in the supplement files which need to be incorporated into the main manuscript file because it is very inconvenient for the reader to keep switching between the files. Summarize the methodology and cite references where required to save space.

*Response: Thank you for your comment. We have re written all the major parts, shifted tables, changed a few figures, added a figure as required and changed the tables as well according to the instructions given below. I hope it will suffice the requirements this time. Please refer to each section response for the details of the changes.*

**Methodology**

- Ethical considerations should be the first paragraph of the methodology.

*Response: Rearranged.*

- A **map of the country/city** pointing out the location of the study sites might be included. Please give information about the study site as well.

*Response: Thank you for your interesting input. A map is now added in the supplement file.*

*[S1 Fig in supplement information]*

- **Study site and enrollment:** Briefly explain the criteria for the classification of dengue patients into suspected, probable, and confirmed cases. Although reference has been provided but brief description of the criteria will facilitate the reader.

*Response; Thank you for this comment. Paragraph rearranged, definitions added.*

*[Page 7; Line: 134-136, page 08; Line: 149-150]*

- Why patients younger than 16 years were excluded from the study? It is an important cohort, and their data could improve the study quality.

*Response: Thank you for your comment. Indeed this age group is very important cohort and the data could have improved the study more. However, this study was conducted in adult wards. To involve that particular age group we would have to extend our study in pediatric wards. This study being conducted in an epidemic situation with high loads in adult wards we did not have the correct manpower or specialty for insight into these cases.*

*That is why this study lacks the scope to profile those cases. Definitely in future there should be a combined study with comparisons of both the age groups.*

- Since dengue has been circulating in Bangladesh for many years, it is possible that many cases were both IgM and IgG positive. They could have been included in the study as well.

*Response: Thank you for your comment. It is very unlikely according to existing national and WHO guidelines that IgM would be circulating in serum for more than 60 days. IgG can be detected for longer times but given the clinical picture and epidemic situation we do consider treating all these cases as dengue. Although ELISA (quantitive) can predict it better with an antibody titre. Unfortunately, in Bangladesh it is not available in government hospital setting.*

**Results**

- Figure 1: Refer to the figure in the text as well.

*Response: Done.*

- **Please compare clinical presentation with similar symptoms among both groups and then compare hemorrhagic manifestations**.

*Response: Thank you for the comment. Rearranged the text.*

*[Page 14; Line: 230-241]*

- **Demographic characteristics: Firstly, discuss general characteristics (like age, gender, etc.). Afterward, characters of different groups and sub-groups might be discussed.**

*Response: Thank you for response. Changes made in the text.*

*[Page 12; Line: 216-217]*

- **Much data is given in the supplement files, however, baseline data related to demographic profile, and clinical presentation might be given in the main manuscript file. It would be very difficult for the reader to keep on switching between the files.**

*Response: Thank you for your feedback. We have transferred Table S9 from supplement as Table 1. Also merged previous S10+11 as Table 2.*

*[Page 13, 15]*

- It has been mentioned that many of the patients from group C were reported outside of Dhaka, however, nothing has been mentioned about how many were reported outside Dhaka in group B

*Response: It was previously mentioned in previous supplement table S9. Added in the main text.*

*[Page 12; Line: 217-219, Table 1]*

- What is meant by service holders? Is it a specific designation in Bangladesh?

*Response: Service holder refers to an individual who is employed by an organization or company to provide services to customers or clients.*

- Throughout the manuscript, IQR has been mentioned as ±N which is not an IQR.

*Response*: *Thank you for your observation, the issue is addressed throughout the manuscript.*

- Whether the com-morbidities were diagnosed or self-reported by the patients? If self-reported by the patients, what about those comorbidities not reported by the patients?

*Response: Co morbidities self-reported by the patients or pre diagnosed by ward physicians. No new comorbidities identified by study physicians and that is also beyond the study objectives.*

- A many places in results, S is written as short for serum which is a non-standard abbreviation. Please write “serum” instead of “S”.

*Response: Thanks, corrected now.*

- Any patient with bacterial infection was excluded from the study so why antibiotic was given to one patient?

*Response: Thank you for your comment, Unfortunately, It is a common practice in many hospitals to use prophylactic antibiotics to prevent secondary infection in severe dengue cases, especially those who need ICU. But the new national guideline strongly discourages this practice. This study highlights the minimization of indiscriminate use of antibiotics in dengue admitted patients in these centers.*

- DENV Serotyping: It is not clear which former infection authors are talking about. Is it "concurrent infections"?

*Response: Thank you for the comment. Sentence rephrased, it now reads as, “Among 86 extracted RNA samples 81 was successfully serotyped out of which DENV-3, DENV-2 and co infection with DENV-2 and 3 were detected in 64 (79 %), 6 (7.4%) and 11 (13.58%) samples respectively (Figure-2).”*

*[Page 20; Line: 276-278]*

- No tests were performed by the authors to confirm primary or secondary infections. Therefore, the authors cannot claim that all infections were primary infections.

*Response: Thank you for your comment. Dengue NS1, anti-dengue IgM and IgG RDT were done as mentioned in the inclusion criteria of the study. We have clarified it in the methodology section in the supplement as well.*

*[Page 8; Line: 147-149; Supplement Page 01; S1.1].*

- Genotyping: Please submit the sequence to Genbank to generate accession numbers which could be mentioned in the manuscript along with percentage similarity.

*Response: Partial C-prM gene sequences of representative five samples were deposited to GenBank database under accession number (OR726315-19) as previously mentioned in methodology*

*[Page 10; Line: 191-192]*

*For clarification and easy access the supplement table 11 now contains a column referring to the GenBank accession numbers hyperlinked to the NCBI website.*

*[Supplement Page 15; Table S11]*

*Further description with percentage similarity was stated in result section, it now reads as,*

*“Regarding DENV-3 serotype, the sequence from Sample no 1(OR726315) showed 99.43% identity with DENV-3 isolates that were reported previously to circulate in Dhaka city during the year 2017 [15]. Sample no 2(OR726316) was found to be identical (100% similarity) with isolates (MW599418.1, MW599415.1) prevalent during 2019 outbreak in Dhaka and Rangpur city of Bangladesh [18]. The sequence from sample no. 17(OR726318) exhibited 100% identity with two GenBank entries (MN922033.1, MN922034.1) of DENV-3 that were isolated in China from Bangladeshi travelers in the year 2019. As for DENV-2 serotype, sample no. 14(OR726319), 99.74% percent identity with three DENV-2 isolates (LC436675.1, LC436674.1, LC436673.1) was observed and all of these were reported to be present in 2017 outbreak in Bangladesh [15].”*

*[Page 22; Line: 310-320]*

**Discussion**

- Please follow a logical order to write results and discussion, starting from the demographic characteristics, followed by clinical signs and symptoms, and finally DENV serotypes and Genotypes.

*Response: Thanks. Yes, we have described your suggested order, enrolment profile, demographics, clinical parameters, laboratory parameters, clinical management and outcome, serotypes and finally genotypes.*

- Write dengue virus serotypes as DENV-1, DENV-2, DENV-3, and DENV-4 throughout the manuscript.

*Response: Done.*

**Table 1**

*Table 1 is now table 3 according to new changes.*

Please avoid non-standard abbreviations like “S. electrolyte”. Write full “Serum”. Correct wherever required. `

*Response: Corrected.*

IQR or interquartile range is a range, therefore, not a single value.

*Response: Thank you for your comment. The table is changed to show mean±SD now.*

*[Page 17]*

Why median values instead of mean values were calculated? Please calculate mean values for each variable to make them more comparable with other studies.

*Response: Thank you for your comment. The table is changed to show mean±SD now.*

*[Page 17]*

- Table 1 should be about the baseline characters of the study population. Signs and symptoms could also be incorporated in Table 1.

*Response: Thank you for your feedback. We have transferred Table S9 from supplement as Table 1. Also merged S10+11 as Table 2,*

*[Page: 13, 15]*

- Label "total" instead of "all"

*Response: Done.*

- Where chi-square was applied as all the variables were quantitative?

*Response: Corrected.*

**Table 3**

- Please mention only significant signs and symptoms in the text. There is no need to make a table for it.

*Response: Table removed from the main draft to the supplement. Breakdown of GI and bleeding manifestations showed.*

*[Supplement file Page 14, Table S10]*

- Please also discuss which hemorrhagic manifestation and GI manifestations were significantly different in these groups.
- *Response: Changed the whole text accordingly.*

*[Page 14; Line: 230-241]*

**Supplement file**

- S.1.1 Overall English needs to be improved. Nonstandard abbreviations like S. for serum must be avoided. Please write in full wherever required.

*Response: Done.*

*[Supplement file page 1, S1.1]*

- Replace “Baseline tests (e.g. CBC, )

*Response: Done.*

*[Supplement file page 1, S1.1]*

Table S10, S11: Characteristics column is not mentioned as n, %. Please correct.

*Response: Done. Merged previous S10+11 as Table 2 as per other comments.*

*[Page 15]*

Write “P-value” as “p-value” throughout the manuscript

*Response: Done*

The symbols used for footnotes in this study are non-standard. Please refer to the journal guidelines.

*Response: Corrected*

Table S10: Please write the duration of fever as mean ± SD

*Response: Done. Merged previous S10+11 as Table 2 as per other comments.*

*[Page 15]*

Age and gender distribution should be part of the demographic profile rather than the clinical presentation table.

*Response: Thanks for your comment. Moved these two in demographic table (Table 1 in main manuscript).*

*[Page 13]*

How primary and secondary infections were differentiated, based upon any test or self-reported by the patients. Self-reported data cannot be relied upon as most of the dengue infections remain subclinical or asymptomatic.

*Response: Thank you for your comment. Primary infection were reported by laboratory evidence of positive NS1 or Anti dengue IgM and secondary infections were declared when patients had Anti dengue IgG positivity done on site by RDT. Due to lack of resources ELISA was not possible.*

*This data was in the wrong place thus the confusion. Thank you for pointing this out. Shifted it in the laboratory section (Table 3 in main manuscript).*

*[Page 17]*

Table S11: What is meant by TT

*Response: Tourniquet test. Previously mentioned in footnote.*

*As per other comments the data now shifted to Table 2 in the manuscript*.

*[Page 15]*

There is no need to write vitals if they remain normal. Write only if there was an increase or decrease due to dengue fever, excluding all other confounding factors. Mention how many patients' vitals were deranged.

*Response: Thank you for your comment. As per case definition, all the group C cases came with compensated shock. No one in group B were in shock. Other vitals like temperature respiratory rate and heart rate were deranged in all explained by dengue fever itself. Thus the data were kept in supplement file, as there’s nothing interesting to highlight here.*

*As per other comments, the data now rearranged in table-2.*

*[Page 15]*

S 2.2. There is no need to give photos of all the gels. Please give one or two properly labeled gel diagram(s) showing different sizes of bands and DNA ladder.

*Response: Thank you for the comment. S2 Fig of supplement information modified according to instructions.*

*[S2 Fig in supplement information]*

Figure S2.2 c and H are not available

*Response: Figure revised as instructed above. Please see S2 Fig of supplement.*

*[S2 Fig in supplement information]*

**References**

- References 1 and 14 are the same

*Response: Thank you for pointing it out. Corrected.*

Other:
**Figure Files:**
While revising your submission, please upload your figure files to the Preflight Analysis and Conversion Engine (PACE) digital diagnostic tool, [https://pacev2.apexcovantage.com](https://pacev2.apexcovantage.com/). PACE helps ensure that figures meet PLOS requirements. To use PACE, you must first register as a user. Then, login and navigate to the UPLOAD tab, where you will find detailed instructions on how to use the tool. If you encounter any issues or have any questions when using PACE, please email us at [figures@plos.org](mailto:figures@plos.org).

*Response: Done.*
 
Data Requirements:
Please note that, as a condition of publication, PLOS' data policy requires that you make available all data used to draw the conclusions outlined in your manuscript. Data must be deposited in an appropriate repository, included within the body of the manuscript, or uploaded as supporting information. This includes all numerical values that were used to generate graphs, histograms etc.For an example see here: <http://www.plosbiology.org/article/info%3Adoi%2F10.1371%2Fjournal.pbio.1001908#s5>.

*Response: The authors confirm that all data underlying the findings are fully available without restriction at figshare, the link is given below-*

*https://figshare.com/articles/dataset/Dengue_Serotype_and_Genotype_2019_Dhaka_outbreak/24686820*

*DOI: https://doi.org/10.6084/m9.figshare.24686820.v1*
Reproducibility:
To enhance the reproducibility of your results, we recommend that you deposit your laboratory protocols in [protocols.io](http://protocols.io/), where a protocol can be assigned its own identifier (DOI) such that it can be cited independently in the future. Additionally, PLOS ONE offers an option to publish peer-reviewed clinical study protocols. Read more information on sharing protocols at <https://plos.org/protocols?utm_medium=editorial-email&utm_source=authorletters&utm_campaign=protocols>

*Response: Thanks for the suggestion.*
