# Supplementary material for: Clinical and laboratory comparison of severe (Group B and C) Dengue cases with molecular characterization from 2019 epidemics in Dhaka, Bangladesh
Source: PLoS Negl Trop Dis. 2024 Nov 25;18(11):e0012686. doi: 10.1371/journal.pntd.0012686 (PMC11627391; doi:10.1371/journal.pntd.0012686)
Supplement: Attachment — Submitted filename: Second Response to reviewers.docx [file pntd.0012686.s007.docx]

**Second response to reviewers**

**PLOS Neglected Tropical Diseases**

**Project Title:** Clinical and laboratory comparison of severe (Group B and C) Dengue cases with molecular characterization from 2019 epidemics in Dhaka, Bangladesh

**Manuscript Number:** PNTD-D-23-01524

1. Methods, Line 147-150 and Discussion Line 416-420. There seems to be some intention to analyze primary infections, and in the discussion “most of the cases in this study was of primary infection as per RDT test reports”. However the relevant results were missing.

Response: Please refer to Table 3 last row for the percentage of secondary infections (5.6%) and the legend shows how it was analyzed. It is obvious that the rest are primary infection (please refer to Table S10 in supplement file) therefore the data was omitted in the main manuscript to avoid redundancy.

[Page 17 line 256]

2. Please also clarify how primary and secondary infections were analyzed.

Response: Please refer to 147-152 and Supplement file text S1.1 and 1.2 for clarification.

“Primary infection were reported by laboratory evidence of NS1 or Anti dengue IgM and secondary infections were declared when patients had Anti dengue IgG positivity...”

[Page 8 line 147-152, supplement file page 1]

3. Table 1. Please use consistent formatting, e.g. “(113) 74.2” should be “113 (74.2)”

Response: Done

[Page 13]

4. Line 233, please change “(p=<0.0001)” to “(p<0.0001)”

Response: Done

[Page 14 line 234]

5. Tables 2, 3 and 4. Most of the outcomes did not have a symmetric distribution and are better presented with median and IQR. Please also update the relevant text accordingly.

Response: Thank you for your input. The data was originally shown as Median and IQR but was changed to mean SD after the reviewer and editor comments last time.

We have changed it again. I hope this will suffice.

N.B: Table 4 was already in median IQR

[Page 15, 17, 19]

6. Table 2, please use consistent decimal places for the percentages

Response: Done

[Page 15]

7. Table 2, please change “>0.9999” to “1.000”

Response: Done

[Page 15]

8. Table 2, please use the same decimal place (1 dp) for mean and SD.

Response: Done

[Page 15]

9. Figure 2, please use 1 decimal place for percentages

Response: Done

[Page 21]

10. Line 418, please present the full term of RDT at first appearance, and if the test was used it should be clearly state in the Methods.

Response: Done. Please refer to 147-152 and Supplement file text S1.1 and 1.2.

[Page 8]

11. Discussion, line 353-359. Please confirm if the mean age was younger than the cited studies only, or exceptionally young? In the latter case, are there any reasons explaining the younger cases in this study?

Response: Thank you for your interesting review. Mean age was younger than the referred studies. However, we may not highlight this young group as exceptional as every study compared to this one has different sample size and may have different inclusion and exclusion criterion. We have pointed out the young group being more vulnerable to the mosquito bite prone environment as they have more outdoor activities.
12. Discussion, if the study population is younger than usual, the impact of a different age distribution should be accessed more carefully when comparing with previous studies (Line 365-378). The authors may consider supplementary analyses stratified by age.
Response: Thank you for comment and excellent suggestion. Unfortunately, we lack enough empirical data like this to analyze the age wise difference. Moreover, the studies we have compared don’t have the data pool shared publicly. Therefore, we cannot make a statement based on this right now. But we agree this warrants a more detailed study on the common age group afflicted by dengue. Thereby we would also get an idea about the age wise severity pattern. Currently this paper lacks the scope to do so. However, we have our data pool shared publicly. In future this might help other researchers when more data will generate with time.
