# Supplementary material for: Clinical and laboratory comparison of severe (Group B and C) Dengue cases with molecular characterization from 2019 epidemics in Dhaka, Bangladesh
Source: PLoS Negl Trop Dis. 2024 Nov 25;18(11):e0012686. doi: 10.1371/journal.pntd.0012686 (PMC11627391; doi:10.1371/journal.pntd.0012686)
Supplement: Attachment — Submitted filename: Third Response to reviewers.docx [file pntd.0012686.s008.docx]

**Third response to reviewers**

**PLOS Neglected Tropical Diseases**

**Project Title:** Clinical and laboratory comparison of severe (Group B and C) Dengue cases with molecular characterization from 2019 epidemics in Dhaka, Bangladesh

**Manuscript Number:** PNTD-D-23-01524
1.      Line 106, please correct the number “1, 01,358”

**Response: Thank you for pointing it out, corrected,**
2.      Tables 2, 3 and 4 have now presented median and a number related to IQR, which is an improvement. As point out by a reviewer in the previous round of review, IQR should be a range. This was well presented for age (IQR = 11-41) in Table 1, but not for other variables.

**Response: Done.**

3.      Tables 2, 3 and 4, please change “(median± IQR)” to “(median, IQR)”. As an example from Table 1, the statistics for age can be presented as “26, 11- 41”

**Response: Done.**
4.      Table 2, please use 1 decimal place consistently for the percentages for all variables

**Response: Done.**

**Additional comment regarding S1 figure:**

Thank you for stating "Map was plotted using Datawrapper website (available at  <https://www.datawrapper.de/_/2ew1H/> ). Basemap shapefile was extracted from OpenStreetMap (after September 2012 is licensed on terms of the Open Database License, "ODbL" 1.0, previously it was licensed CC-BY-SA 2.0). For more copyright related info: <https://www.openstreetmap.org/copyright>". 
Unfortunately this source for FIGURE S1 is copyrighted and is not compatible with Creative Commons Attribution 4.0 International (CC BY 4.0) License used by PLOS journals.
We cannot publish proprietary or copyrighted maps (e.g. Google Maps, Mapquest) and the terms of use for your map base layer must be compatible with our CC BY 4.0 license.

…… If your map was obtained from a copyrighted source please amend the figure so that the base map used is from an openly available source.

Alternatively, please provide explicit written permission from the copyright holder granting you the right to publish the material under our CC BY 4.0 license

**Response: The OSM basemap was convert from CC-BY-SA to ODbL and is no longer under CC-BY-SA. ODbL is an open license standard and images produced from ODbL data are compatible with CC-BY 4.0, requiring only attribution.**

**Additionally, permission was taken from the concerned to publish the figure in PlosNTD papers (attached herewith).**
