# Supplementary material for: Clinical and laboratory comparison of severe (Group B and C) Dengue cases with molecular characterization from 2019 epidemics in Dhaka, Bangladesh
Source: PLoS Negl Trop Dis. 2024 Nov 25;18(11):e0012686. doi: 10.1371/journal.pntd.0012686 (PMC11627391; doi:10.1371/journal.pntd.0012686)
Supplement: Attachment — Submitted filename: Fourth Response to reviewers.docx [file pntd.0012686.s009.docx]

**Fourth response to reviewers**

**PLOS Neglected Tropical Diseases**

**Project Title:** Clinical and laboratory comparison of severe (Group B and C) Dengue cases with molecular characterization from 2019 epidemics in Dhaka, Bangladesh

**Manuscript Number:** PNTD-D-23-01524

Thank you very much for the appreciation and considering acceptance of the manuscript (Clinical and laboratory comparison of severe (Group B and C) Dengue cases with molecular characterization from 2019 epidemics in Dhaka, Bangladesh", Manuscript Number: PNTD-D-23-01524). The review process has been highly constructive. We tried utmost to modify the manuscript according to instructions of several reviewers. Please find your latest corrections as below,

1.      ***Line 106, please correct the number “1,01,354”. Was that 1 million or 100 thousand?haracterization from 2019 epidemics in Dhaka, Bangladesh".***

**Response: The number is one hundred one thousand, three hundred fifty-four. Please see the clarification at line 106-7, page 5-6.**

2.      ***Table 2, please correct the numbers “20, 0” for the pulse pressure of Group C***

**Response: The IQR is 20-20 as per calculation for the pulse pressure in group C. Maybe due to a narrow range in hypotensive patients.**

3.      ***Table 3, please add a comma in “50001900- 8100”***

**Response: Thank you for pointing out this typo, corrected the dataset.**

4.      ***Table 3, please correct the negative values (e.g. N:L ratio, AST and others)***

**Response:  Thank you for pointing out this error, corrected the errors in IQR range calculation.**

4.      ***Tables 3 and 4, please harmonize the decimal places for the numbers presented***

**Response:**  **The numbers were in one decimal places as per response from the previous reviewer. If you need a round up value without the decimals please clarify. Harmonized as much as possible.**

We would highly appreciate the clarification for the above comments and looking forward to getting this important manuscript published soon. As the submission date is on 16^th^ November, 2024, we would appreciate your response as soon as possible.

Kindest regards

Dr. Fazle Rabbi Chowdhury

Bangabandhu Sheikh Mujib Medical University

Dhaka, Bangladesh
